# Photoinduced Electron Transfer from a 1,4,5,6-Tetrahydro Nicotinamide Adenine Dinucleotide (Phosphate) Analogue to Oxidized Flavin in an Ene-Reductase Flavoenzyme

**DOI:** 10.1021/acs.jpclett.3c00176

**Published:** 2023-03-27

**Authors:** Magnus Speirs, Samantha J. O. Hardman, Andreea I. Iorgu, Linus O. Johannissen, Derren J. Heyes, Nigel S. Scrutton, Igor V. Sazanovich, Sam Hay

**Affiliations:** † Manchester Institute of Biotechnology and Department of Chemistry, Faculty of Science and Engineering, The University of Manchester, 131 Princess Street, Manchester M1 7DN, United Kingdom; ‡ Central Laser Facility, Research Complex at Harwell, Science and Technology Facilities Council, Harwell Oxford, Didcot OX11 0QX, United Kingdom

## Abstract

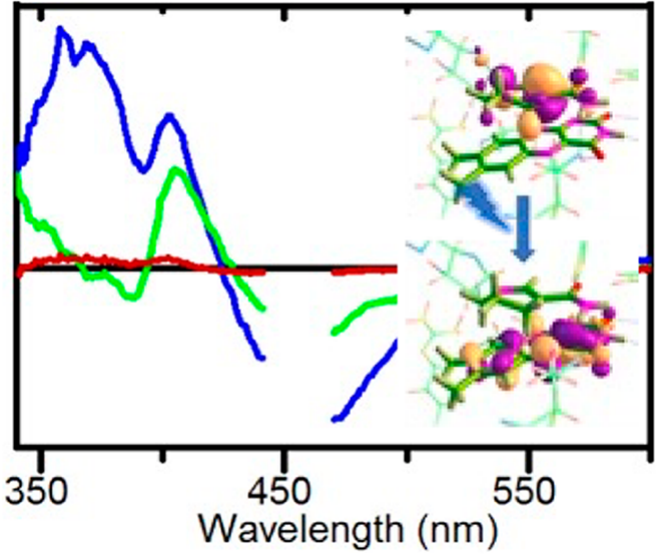

Recent reports have described the use of ene-reductase flavoenzymes to catalyze non-natural photochemical reactions. These studies have focused on using reduced flavoenzyme, yet oxidized flavins have superior light harvesting properties. In a binary complex of the oxidized ene-reductase pentaerythritol tetranitrate reductase with the nonreactive nicotinamide coenzyme analogs 1,4,5,6-tetrahydro NAD(P)H, visible photoexcitation of the flavin mononucleotide (FMN) leads to one-electron transfer from the NAD(P)H_4_ to FMN, generating a NAD(P)H_4_ cation radical and anionic FMN semiquinone. This electron transfer occurs in ∼1 ps and appears to kinetically outcompete reductive quenching from aromatic residues in the active site. Time-resolved infrared measurements show that relaxation processes appear to be largely localized on the FMN and the charge-separated state is short-lived, with relaxation, presumably via back electron transfer, occurring over ∼3–30 ps. While this demonstrates the potential for non-natural photoactivity, useful photocatalysis will likely require longer-lived excited states, which may be accessible by enzyme engineering and/or a judicious choice of substrate.

Flavoenzymes are ubiquitous in nature, where they are utilized in a large variety of different redox reactions essential to life.^1,2^ They can achieve this due to their four main oxidation states, which through steric and electrostatic effects from the enzyme allow a wide range of reductive and oxidative potentials to be achieved, leading to diverse and interesting redox chemistry. Consequently, flavin cofactors play a central role in biological redox chemistry. The spectral properties of the different oxidation states vary, and both oxidized and reduced flavins can also be utilized as photocatalysts. Flavin adenine dinucleotide (FAD) is utilized as a photosensitizer by two of the three known classes of natural photoenzymes,^3,4^ DNA photolyase involving photoexcitation of the neutral radical FADH^•^ and fatty acid photolyase (FAP) involving photoexcitation of oxidized FAD. Recent work by the Hyster group has shown that “light-independent” flavin-dependent “ene” reductases such as pentaerythritol tetranitrate reductase (PETNR) can become more substrate promiscuous after photoexcitation of the flavin.^5−7^ These authors have mainly focused on photoexcitation of the reduced flavin. However, oxidized flavins have a higher extinction coefficient in the visible region so can be much more efficiently excited by natural light. Photoexcitation of oxidized flavoenzymes would offer a route to the oxidation of new substrates that are not efficiently oxidized by the ground state “dark” flavin cofactor. However, reductive quenching of the photoexcited flavin has been shown to efficiently outcompete productive photoreduction,^8−13^ which makes it challenging to catalyze non-natural photooxidation reactions using flavoenzymes.

In this study, we investigate the photochemistry of oxidized PETNR with the nonreactive nicotinamide adenine dinucleotide (phosphate) [NAD(P)H] coenzyme analogues 1,4,5,6-tetrahydro-NAD(P)H [NAD(P)H_4_ (Figure 1)]. NAD(P)H_4_ is nonreactive because two-electron oxidation is not stabilized by aromatization [which is the case in natural coenzymes NAD(P)H (Figure 1a)]. However, one-electron oxidation of NAD(P)H has also been observed with strong oxidants^14,15^ and during photoexcitation of the nicotinamide,^16,17^ which suggests that NAD(P)H_4_ may be able to act as a one-electron reductant. If so, we hypothesize that photoexcitation of the PETNR–NAD(P)H_4_ binary complex may generate the NAD(P)H_4_ cation radical via electron transfer (eT) from NAD(P)H_4_ to flavin mononucleotide (FMN) (flavin). Time-resolved visible absorption spectroscopy (TRVis) and time-resolved infrared absorption spectroscopy (TRIR) were used to investigate the short-lived changes to the electronic and nuclear structure of NAD(P)H_4_-bound PETNR after photoexcitation of the intrinsic FMN cofactor. Density functional theory (DFT) and time-dependent DFT (TD-DFT) were then employed to model and confirm the identity of the intermediates. We show that it is possible to generate a short-lived FMN semiquinone upon visible photoexcitation.

**Figure 1:**
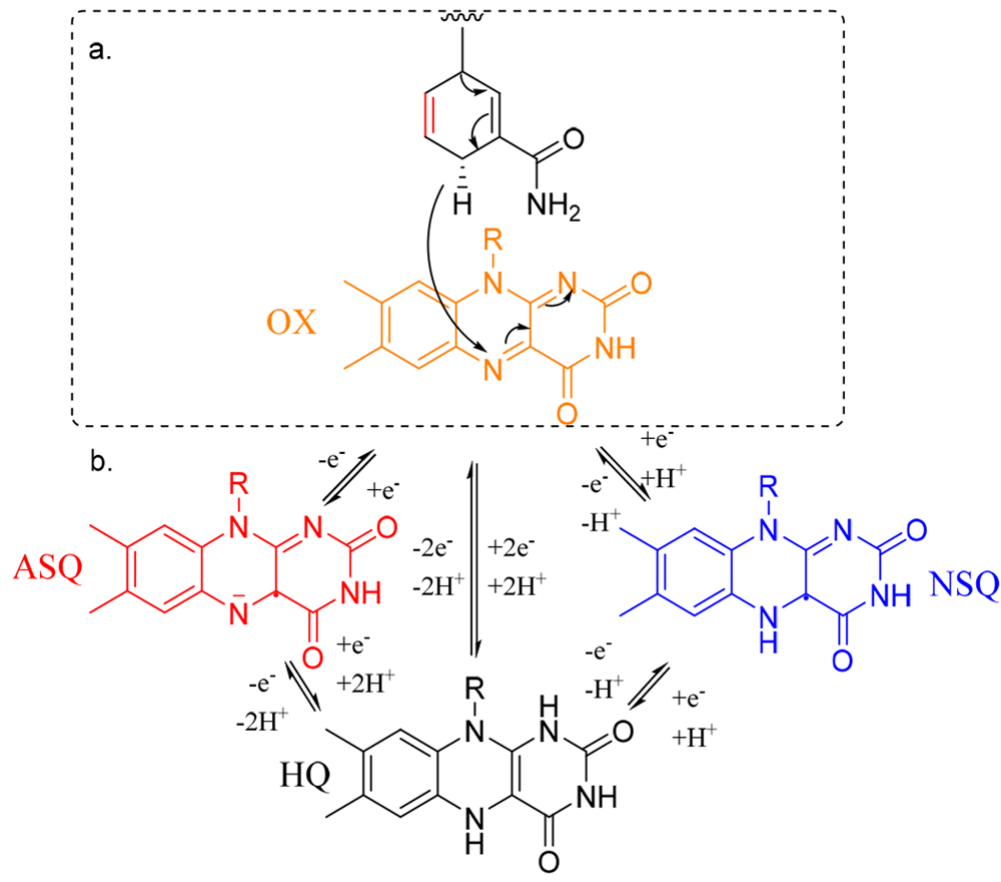
(a) Mechanism of the reductive half-reaction of PETNR, involving hydride transfer from NAD(P)H to FMN. The bond that is colored red is saturated in NAD(P)H_4_. (b) Structures of flavin in the oxidized (Ox, orange), reduced (HQ, black), anionic semiquinone (ASQ, red), and neutral semiquinone (NSQ, blue) oxidation states.

We have previously shown that incorporation of different stable “heavy” isotopes (^2^H, ^13^C, and ^15^N) in the protein scaffold and/or the FMN cofactor of PETNR can be used to experimentally probe changes in the protein and/or flavin upon photoexcitation.^13^ So that peaks in the TRIR spectra can be more clearly assigned, we have used two isotopologues of PETNR in this study: FMN bound [^15^N]PETNR (denoted ^15^N PETNR:L FMN) and [^2^H,^13^C,^15^N]FMN bound [^2^H,^13^C,^15^N]PETNR (denoted H PETNR:H FMN).

Initially, TRVis measurements were performed on ^15^N PETNR:L FMN samples with and without NADH_4_ and NADPH_4_ bound in buffered H_2_O and D_2_O solutions (static absorption spectra shown in Figure S1). After excitation of the FMN at 450 nm, spectra were recorded between 0.2–200 ps and 0.2 ps to 1 ns, for samples with and without bound NAD(P)H_4_, respectively. These transient spectral data (Figure 2A–E) were fitted globally using three variable components (Figure 2F–J, and normalized to the maximum intensity bleach feature in Figure S2). The fitted lifetimes show no substantial differences between H_2_O and D_2_O buffer conditions (Table 1), suggesting there is no measurable solvent isotope effect.

**Figure 2:**
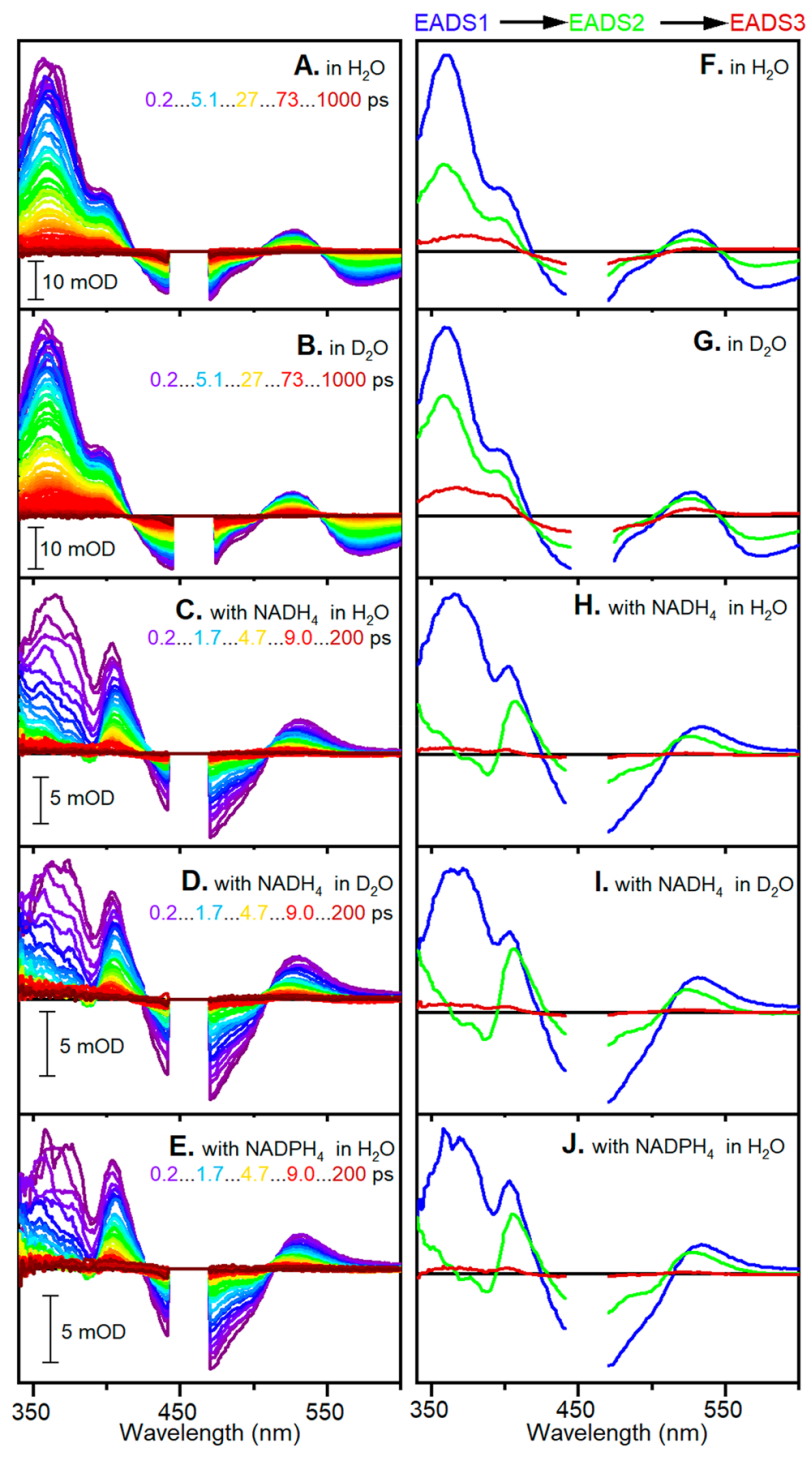
(A–E) Difference spectra at selected time points and (F–J) EADS resulting from global analysis of TRVis data using a sequential model of three sequentially interconverting components for ^15^N PETNR:L FMN in H_2_O and D_2_O, ^15^N PETNR:L FMN:NADH_4_ in H_2_O and D_2_O, and ^15^N PETNR:L FMN:NADPH_4_ in H_2_O.

**Table 1:** Fitted Lifetimes from Global Analysis of the TRVis Data in Figure 2 Using a Model with Three Free Components

sample	τ_1_ (ps)	τ_2_ (ps)	τ_3_ (ps)
^15^N PETNR:L FMN in H_2_O	4.8	21.0	81.8
^15^N PETNR:L FMN in D_2_O	5.0	18.9	86.3
^15^N PETNR:L FMN:NADH_4_ in H_2_O	0.64	2.2	73.3
^15^N PETNR:L FMN:NADH_4_ in D_2_O	0.81	1.9	118.3
^15^N PETNR:L FMN:NADPH_4_ in H_2_O	0.67	2.1	58.7

For the samples lacking NAD(P)H_4_, the spectral features all appear to originate from the FMN and include the ground state bleach at ∼460 nm and stimulated emission at ∼575 nm, with excited state absorption between them. The first two evolution-associated difference spectra (EADS) are very similar in shape (Figure S2) and may report on two subpopulations of the protein with different structural conformations and lifetimes.^18−20^ When NAD(P)H_4_ is bound, there is an additional broad feature in the ground state absorption spectral features (Figure S1) arising from the charge transfer complex formed between the FMN and reduced nicotinamide moiety of NAD(P)H_4_.^21,22^ Excitation of this feature at 550 nm leads to no observable TRVis features (Figure S3). Upon excitation at 450 nm, there is also no longer any significant stimulated emission observed in the TRVis data, which implies that virtually all of the excited state decay is nonradiative. The addition of NAD(P)H_4_ also leads to a considerable shortening of all three excited state lifetimes (Table 1), which suggests other, more efficient relaxation pathways are present when NAD(P)H_4_ is bound. One of these pathways must be very rapid because even in the earliest spectra there is very little stimulated emission. A likely excited state relaxation process is reductive quenching, involving eT to the FMN. This will result in the formation of either anionic or neutral FMN semiquinones, both of which have distinct visible absorption features. The neutral radical has a broad absorption band between 450 and 650 nm, while the anionic radical has sharp intense features at approximately 364 and 402 nm.^23^ These features have successfully been used to follow photoinduced kinetics in BLUF domains^24^ and photolyases.^25^ The TRV is data (Figure 2H–J) do not show any of the broad positive absorption band above 450 nm that would be expected from a neutral radical species, and there is a sharp positive feature at ∼406 nm, consistent with this arising from anionic FMN absorption. This suggests that photoexcitation of the FMN leads to generation of the anionic FMN semiquinone via one-electron reduction. The spectral properties of the NAD(P)H_4_ cation radical have not been reported, but we do not expect these species to have absorption in the visible region.

To further characterize this photochemistry, TRIR measurements were performed on both isotopologues of PETNR with bound NAD(P)H_4_ (static FTIR spectra shown in Figure S4)_._ Spectra were recorded between 0.4 ps and 12 ns after excitation at 450 nm. The raw data shown in Figures S5–S8 were fitted globally (EADS in Figure 3 and fitted lifetimes in Table 2) using three variable components and one fixed component to represent any persistent structural changes that are not apparent in the sets of TRVis data, which report on only changes in the electronic states of the flavin. It is immediately apparent when comparing these TRIR data to those previously collected in the absence of the coenzyme^26^ that the addition of coenzyme changes both the kinetics of the processes that occur after excitation and the nature of the relaxation steps (Table 2). The most obvious difference upon coenzyme binding is the significant reduction in excited state lifetime, in agreement with the TRVis data (Table 1). It is likely that in both TRVis and TRIR data sets the first lifetime corresponds to eT and the second to back-eT. However, the third fitted TRIR lifetime of ∼10 ps is much faster than the lifetime of ∼80 ps from the TRVis data, suggesting the spectral signals have different origins. The slower lifetime component from the TRVis data is of extremely low intensity and may result from a very small proportion of free FMN, which is unlikely to affect the fitting. The faster lifetime component from the TRIR data has more significant intensity and must result from a structural change, perhaps to the NAD(P)H_4_, which does not impact the visible absorption features (FMN electronic state). In both TRVis and TRIR data, there do not appear to be any differences in the fitted lifetimes or spectral features between the two coenzyme analogues, suggesting that the NAD(P)H_4_ adenine (phosphate) tail does not play a significant role in these relaxation processes.

**Table 2:** Fitted Lifetimes from Global Analysis of the TRIR Data in Figure 3 Using a Model with Three Free Components and One Constant

sample (all in D_2_O)	τ_1_ (ps)	τ_2_ (ps)	τ_3_ (ps)
^15^N PETNR:L FMN^a^	4.6	19.6	92.3
H PETNR:H FMN^a^	5.1	21.8	98.2
^15^N PETNR:L FMN:NADH_4_	1.4	2.4	14.8
^15^N PETNR:L FMN:NADPH_4_	1.3	2.6	16.0
H PETNR:H FMN:NADH_4_	1.5	1.9	9.5
H PETNR:H FMN:NADPH_4_	1.2	2.6	9.5

a Data from ref (26).

**Figure 3:**
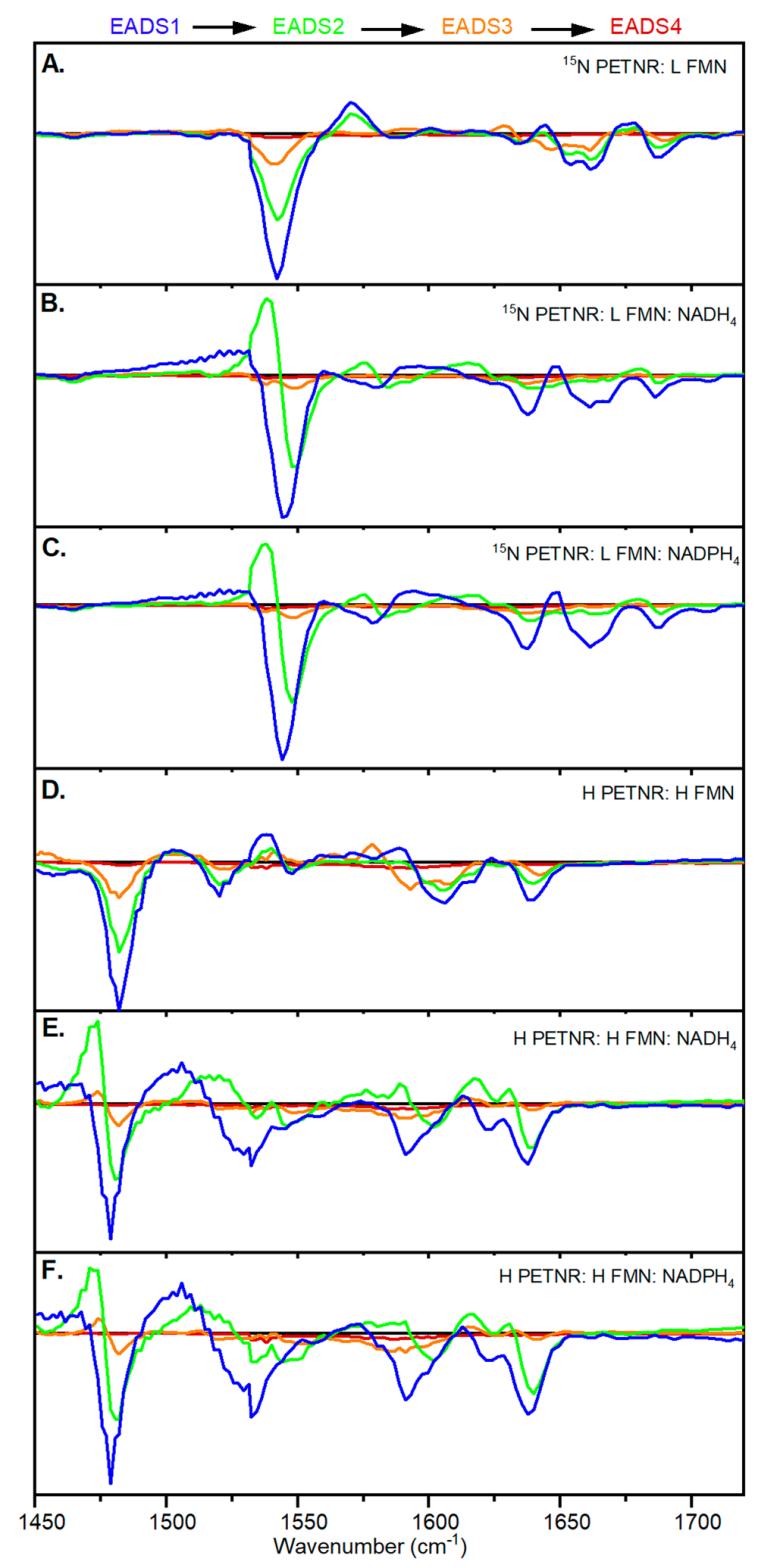
EADS resulting from global analysis of TRIR data shown in Figures S4–S7 using a sequential model of four interconverting components: (A) ^15^N PETNR:L FMN (data from ref (26)), (B) ^15^N PETNR:L FMN:NADH_4_, (C) ^15^N PETNR:L FMN:NADPH_4_, (D) H PETNR:H FMN (data from ref (26)), (E) H PETNR:H FMN:NADH_4_, and (F) H PETNR:H FMN:NADPH_4_.

The EADS that show the TRIR spectral evolution are shown in Figure 3 and normalized to the maximum intensity bleach feature in Figure S9. The EADS arising from samples containing NAD(P)H_4_ show a number of extra features compared to those of the coenzyme-free samples; most obviously a new, narrow positive feature to the low-energy side (1538 and 1472 cm^–1^ for “light” and “heavy” FMN, respectively) of the major ground state bleach appears in the transition from EADS1 to EADS2 (∼1.4 ps) and then disappears with the appearance of EADS3 (∼2.4 ps). None of the features that appear in the coenzyme-bound spectra appear to originate from the protein itself (see Figure S10). It is possible that there may be some spectral contribution from the NAD(P)H_4_ in EADS2 (see Figure S11). Various studies of flavins and flavoproteins have identified a narrowing and downshifting of the C=N feature, which occurs at ∼1550 cm^–1^ in the neutral ground state originating from a radical species. Features at 1528 cm^–1^ (methylated FAD and FMN),^27^ 1532 cm^–1^ (BLUF),^28^ 1535 cm^–1^ (cryptochrome),^29^ 1530 cm^–1^ (LOV domain),^29^ and 1535 cm^–1^ (BLUF)^30^ have been previously assigned to the neutral semiquinone (FADH neutral semiquinone), while the anionic semiquinone (FAD anionic semiquinone) has apparently been observed at 1515 and 1521 cm^–1^ (both in BLUF domains).^30,31^ Measurements of neutral and anionic FAD semiquinones in glucose oxidase showed an only small difference in the time-resolved IR spectra.^27^ Definitive assignment of the feature we observe in the IR region is therefore not trivial. However, the time scales of the spectral feature’s formation and loss are more consistent with an eT to form the anionic semiquinone than the additional proton transfer needed to form the neutral semiquinone. This assignment is consistent with the tentative assignment of FMN anionic semiquinone observed in the TRVis data sets.

Although both spectroscopic techniques suggest an anionic semiquinone is formed by rapid eT from the NAD(P)H_4_ to the FMN, neither could provide definitive assignment, so we also investigated the photoexcitation event using TD-DFT calculations of an active site “cluster” model. The 175-atom model is based on Protein Data Bank (PDB) entry 3KFT and contains a truncated FMN, NAD(P)H_4_, and first-sphere residues that form key interactions (H-bond, electrostatic and/or steric) with the FMN and/or NAD(P)H_4_ (Figure 4) Additional information is given in the Supporting Information. The model was geometry optimized in the ground state and first excited singlet state using the M06-2x/6-31G(d,p) level of theory with dispersion correction and implicit solvation with a water model to mimic the relatively solvent-exposed nature of PETNR’s active site.

**Figure 4:**
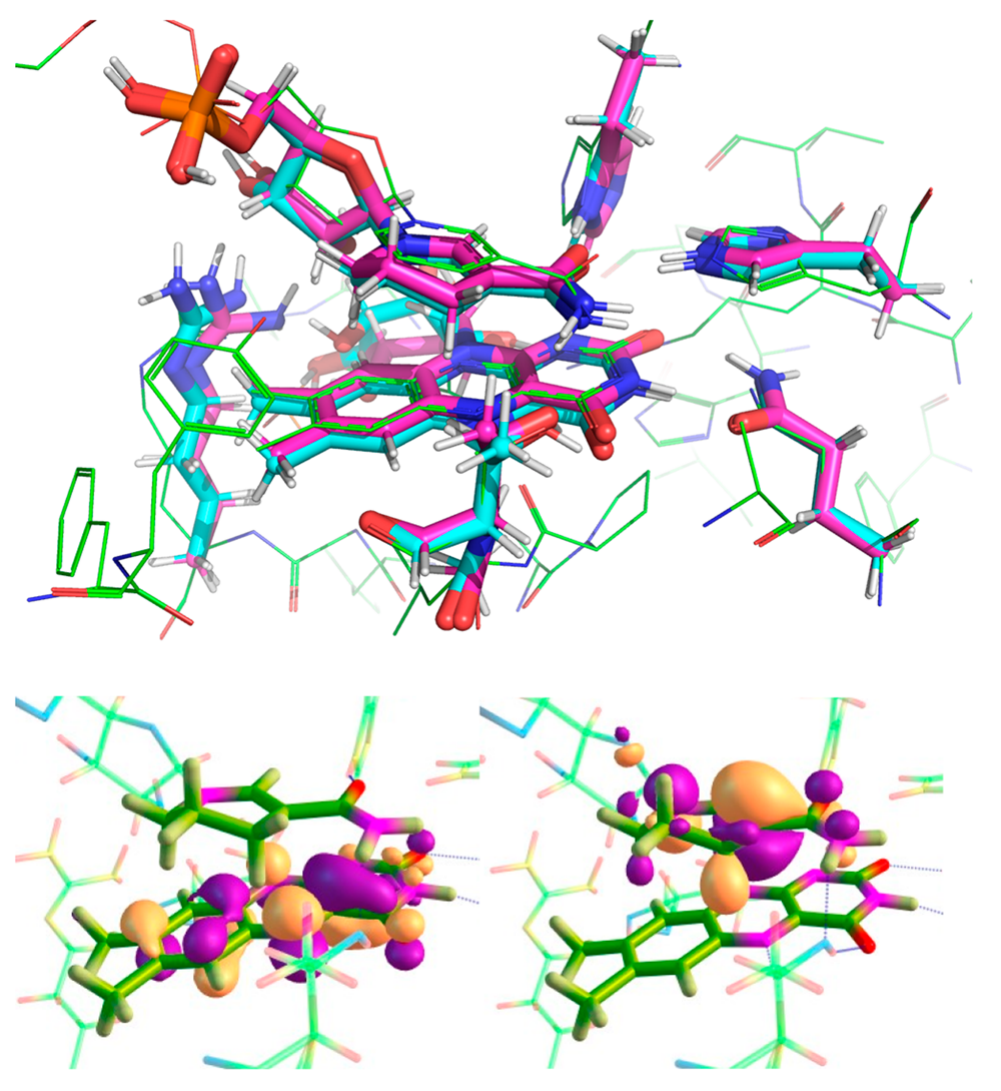
Overlay of the geometry-optimized DFT active site “cluster” model in the ground state (cyan) and first singlet excited state (magenta) with the X-ray crystal structure of PDB entry 3KFT (green) (top). Molecular orbital analysis of the geometry-optimized TD-DFT active site cluster model showing the electron-accepting orbital centered on the FMN (left) and the electron-donating orbital centered on the NAD(P)H_4_ (right) (bottom).

The TD-DFT model shows a vertical S_0_ → S_1_ excitation energy of 281.6 kJ mol^–1^ (425 nm), which is in reasonable agreement with the absorbance maxima of the PETNR–NADH_4_ binary complex (see Figure S1). The model also shows a charge transfer of ∼0.68 electron (e) from the NAD(H)_4_ to FMN upon comparison of single-point TD-DFT calculations to the ground state between the two transitions (Tables S1 and S2). This is consistent with the observation of a charge transfer complex formed upon binding of NAD(H)_4_ to PETNR.^32^ When the first excited state is geometry-optimized, the charge transfer increases to ∼0.89 e and the near unity magnitude of this value suggests that photoexcitation leads to a single eT from NAD(P)H_4_ to FMN, which can be stabilized by molecular rearrangement in the excited state. Geometry optimization lowers the S_1_ excited state by 62.2 kJ mol^–1^ (Table S1). The charge transfer occurs between a donating π-molecular orbital centered on the NAD(P)H_4_ nicotinamide and an accepting π-molecular orbital delocalized over the FMN isoalloxazine ring system. This is observed in the optimized and single-point calculations (Figure 4 and Table S2). No proton transfers are observed in the geometry-optimized excited state, consistent with the formation of the NAD(P)H_4_ cation radical and anionic FMN semiquinone radical proposed above. Comparison of the S0 and S1 geometries shows only minor changes in geometry, consistent with the geometrically constrained and preorganized (charge transfer) nature of the active site in the ground state.

We show that in a binary complex of PETNR with the nonreactive NAD(P)H_4_ coenzyme, visible photoexcitation of the FMN leads to one-electron transfer from the NAD(P)H_4_ to FMN to generate an NAD(P)H_4_ cation radical and the anionic FMN semiquinone. This eT occurs in ∼1 ps and appears to, at least in part, kinetically outcompete reductive quenching from aromatic residues in the active site. TRIR measurements show that relaxation processes appear to be largely localized to the FMN and the charge-separated state is short-lived, with relaxation, presumably by back-eT, occurring over ∼2–3 ps. These experiments may be a useful tool for the enzyme engineer when optimizing or troubleshooting the design of new flavoenzyme-catalyzed photochemical reactions.

While they have superior absorbance cross sections, it appears that it will be challenging to use oxidized ene-reductase enzymes (cf. reduced enzymes) as non-natural photoenzymes. While charge separation (forward eT) is favorable with appropriate substrates that can bind in the proximity of the flavin (i.e., with a short eT distance and a rapid eT that can outcompete reductive quenching), rapid relaxation of the charge-separated state(s) via back-eT will lead to nonproductive charge recombination. However, there are a number of strategies that could be used to overcome this challenge. Substrates could be selected such that they undergo rapid photochemical rearrangement, such as the decarboxylation observed in FAP^4,33^ or the recent example of substrate photoswitching in a flavoenzyme.^34^ Alternatively, enzyme engineering and/or careful substrate selection could be used to tune the flavin/substrate reduction potentials such that the back-eT reaction occurs in the Marcus inverted region,^35^ which would slow back-eT and thus increase the excited state lifetime. A recent report of a high-throughout strategy for screening ene reductase photocatalysis in directed evolution experiments^36^ provides one approach to screening. Our work suggests that selecting slow back-eT may be more important than optimizing charge separation, so any screening strategy should account for this.

## Experimental Section

Sample preparation and TRIR and TRVis measurements and analysis were performed as described previously,^26,37^ and this information is also included in the Supporting Information. Samples contained approximately 0.35 mM PETNR:FMN for TRVis measurements and 1.0 mM PETNR:FMN for TRIR measurements. NAD(P)H_4_ was added to samples to a final concentration of 10 mM. All samples contained 50 mM potassium phosphate (pH or pD 7.0), and the reactions were performed in D_2_O (all TRIR, TRVis) or H_2_O (TRVis as indicated) at room temperature.

Active site DFT cluster models contained truncated NADH_4_ and FMN molecules and six amino acids (His181, His184, Arg324, Gln100, Leu25, and Thr26) to give a model containing 175 atoms in total. The positions of all β-carbons on the amino acids except Thr26, the phosphorus atom in the truncated NADH_4_, and the terminal oxygen atom on the truncated FMN were fixed to the positions found in the X-ray crystal structure of NADH_4_-bound PETNR (PDB entry 3KFT, seven atoms fixed in total). Calculations were performed at the M06-2x/6-31G(d,p) level of theory with the D3 version of Grimme’s dispersion with Becke–Johnson damping^38^ and a polarizable continuum model for water. In all cases, the multiplicity was 1 and TD-DFT calculations were performed with 10 singlet excited states (*n* = 10) and root = 1 to describe the first singlet excited state. Charges were computed using the natural bond order method. All TD-DFT geometry optimization and frequency calculations were performed using Gaussian 16 revision C.01, and all other calculations performed using Gaussian 16 revision D.01.^39^ Additional information and model coordinates are included in Tables S1–S3.

## Data Availability

All data supporting this study are provided in the main text and the Supporting Information.

## Supporting Information Available

Additional experimental and computational details and analysis, including DFT model Cartesian coordinates (PDF)
Transparent Peer Review report available (PDF)
