# Supplementary material for: Photoinduced Electron Transfer from a 1,4,5,6-Tetrahydro Nicotinamide Adenine Dinucleotide (Phosphate) Analogue to Oxidized Flavin in an Ene-Reductase Flavoenzyme
Source: J Phys Chem Lett. 2023 Mar 27;14(13):3236–42. doi: 10.1021/acs.jpclett.3c00176 (PMC10084465; doi:10.1021/acs.jpclett.3c00176)
Supplement: Supplementary file 1 — jz3c00176_si_001.pdf [file jz3c00176_si_001.pdf]

Photoinduced Electron Transfer from a 1,4,5,6-Tetrahydro Nicotinamide Adenine Dinucleotide (Phosphate) Analog to Oxidized Flavin in an Ene-Reductase Flavoenzyme

Magnus Speirs[1], Samantha J.O. Hardman[1*], Andreea I. Iorgu[1], Linus O. Johannissen[1], Derren J. Heyes[1], Nigel S. Scrutton[1], Igor V. Sazanovich[2], Sam Hay[1*]

[1] Manchester Institute of Biotechnology and Department of Chemistry, Faculty of Science and Engineering, The University of Manchester, 131 Princess Street, Manchester M1 7DN, United Kingdom.

[2] Central Laser Facility, Research Complex at Harwell, Science and Technology Facilities Council, Harwell Oxford, Didcot OX11 0QX, United Kingdom.

Samantha.Hardman@manchester.ac.uk; Sam.Hay@manchester.ac.uk

Additional experimental methods

Materials. All reagents were of analytical grade and were purchased from Sigma-Aldrich (Dorset, UK), except for NADH and NADPH, which were obtained from Melford Laboratories (Chelsworth, U.K.). All isotopically enriched compounds (^2H_2O, $^{15}NH_4Cl$ and $^2H_7$,$^{13}C_6$-D-glucose) used for ^2H,^{13}C,^{15}N-labeled PETNR overexpression were obtained from Goss Scientific Ltd. (Crewe, U.K.).

Overexpression and purification of PETNR isotopologues, and sample preparation. A pET21a plasmid encoding a C-terminal His_6-tagged (C-His_6) PETNR gene was used for the production of all PETNR isotopologues. All expression and purification steps are covered detail elsewhere.[1, 2]
All protein samples were expressed with ^{15}N labeling, as this allowed further structural quality control using NMR spectroscopy[1, 3]

Preparation of deuterated and reduced coenzymes. 1,4,5,6-tetrahydro analogues of NADPH ($NADPH_4$) and NADH ($NADH_4$) were synthesised by reduction of NADPH and NADH, respectively with hydrogen using palladium-activated charcoal, and purified as described previously.[4]

TRVis spectroscopy. TRVis spectroscopy was performed using a Ti:sapphire amplifier system (Newport Spectra Physics, Solstice Ace) producing 800 nm pulses at 1 kHz with 100 fs pulse duration. A TOPAS Prime optical parameter amplifier with associated NirUVis unit was pumped with a portion of the output of the amplifier to generate the pump beam at 450 nm with 0.4 uJ pulse energy. A broadband ultrafast pump-probe transient absorbance spectrometer 'Helios' (Ultrafast Systems LLC) was used to collect data over a 3 ns time window with a time resolution of around 250 fs. The probe beam consisted of a white light continuum generated in a CaF_2 crystal and absorbance changes were monitored between 340 and 600 nm. Measurements were performed in a 2 mm pathlength magnetically stirred quartz cell. The pre-excitation data were subtracted before the analysis and spectral chirp corrected for.

TRIR spectroscopy. TRIR spectroscopy was performed using the TRMPS set-up of the ULTRA-LIFEtime system at the Central Laser Facility, STFC, Rutherford Appleton Laboratory, UK. This uses a 100 kHz ultrafast laser based on a custom dual Yb:KGW system (Pharos, Light Conversion). Samples were contained between two CaF_2 windows, separated by a teflon spacer to give a pathlength of approximately 50 µm. The excitation beam was set at 450 nm with 0.8 uJ at 1 kHz repetition rate, and the polarization of it was set at magic angle with respect to the IR probe beam. The sample holder was

rastered to avoid sample damage. Data were collected for approximately 30 minutes per dataset. Difference spectra were generated relative to the ground state in the spectral window 1450-1720 cm^{-1} at time delays ranging between 0.4 ps and 12 ns. Pixel to wavenumber calibration was performed using a polystyrene standard.

Data analysis. Global analysis was performed using the software Glotaran to obtain an evolution associated difference spectra (EADS).[5] This procedure reduces the matrix of change in absorbance as a function of time and wavelength, to a model of one or more exponentially decaying time components, each with a corresponding difference spectrum (EADS). All datasets were fitted to a sequential, unbranched model.

Additional experimental data:

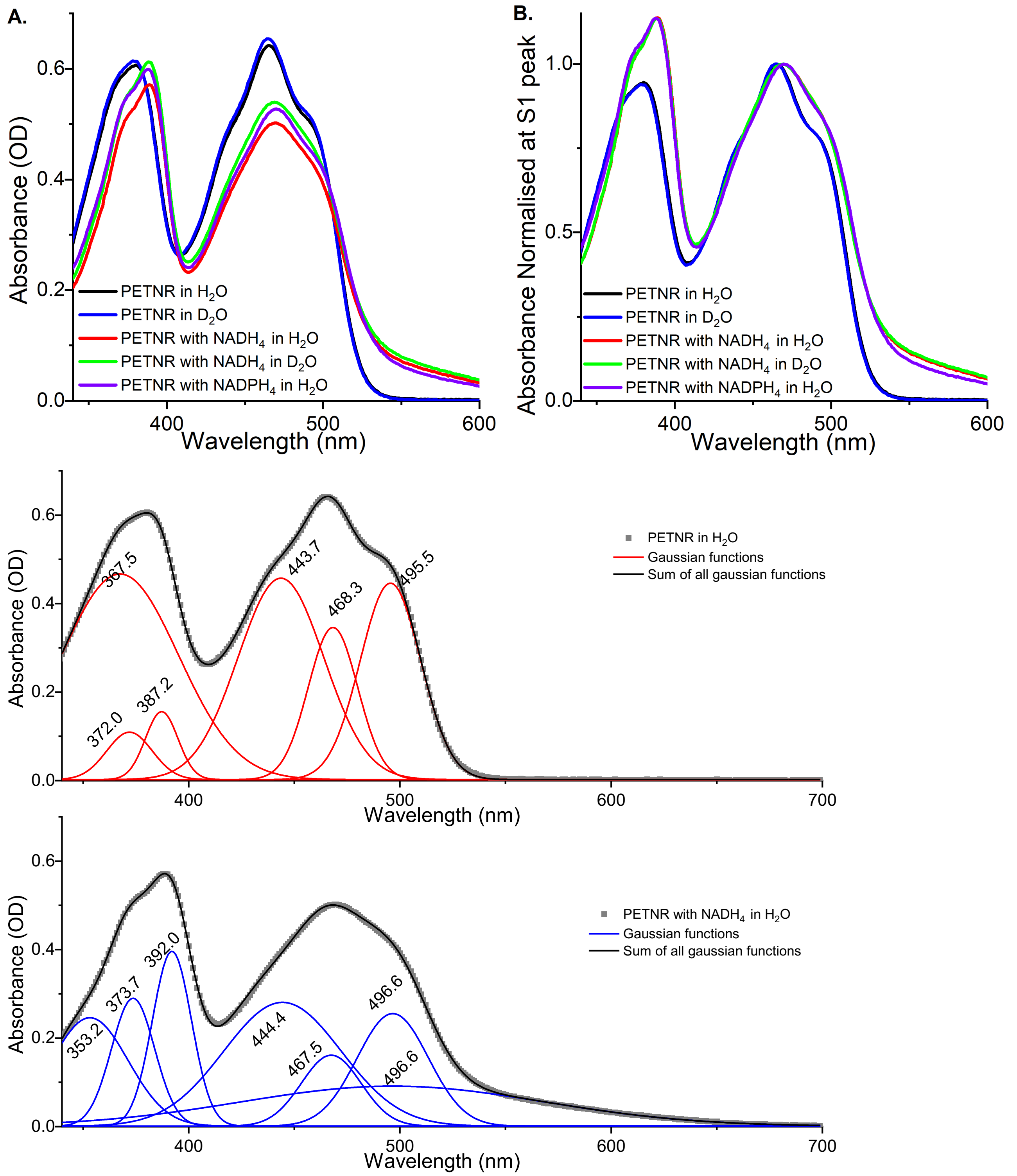

Figure S1. *Top,* Visible absorbance spectra recorded on the samples used in TRVis measurements (**A**) and normalized to the S1 absorbance peak at ~460 nm (**B**). *Bottom,* The spectra of PETNR and $NADH_4$-bound PETNR deconvoluted using a sum of Gaussians.

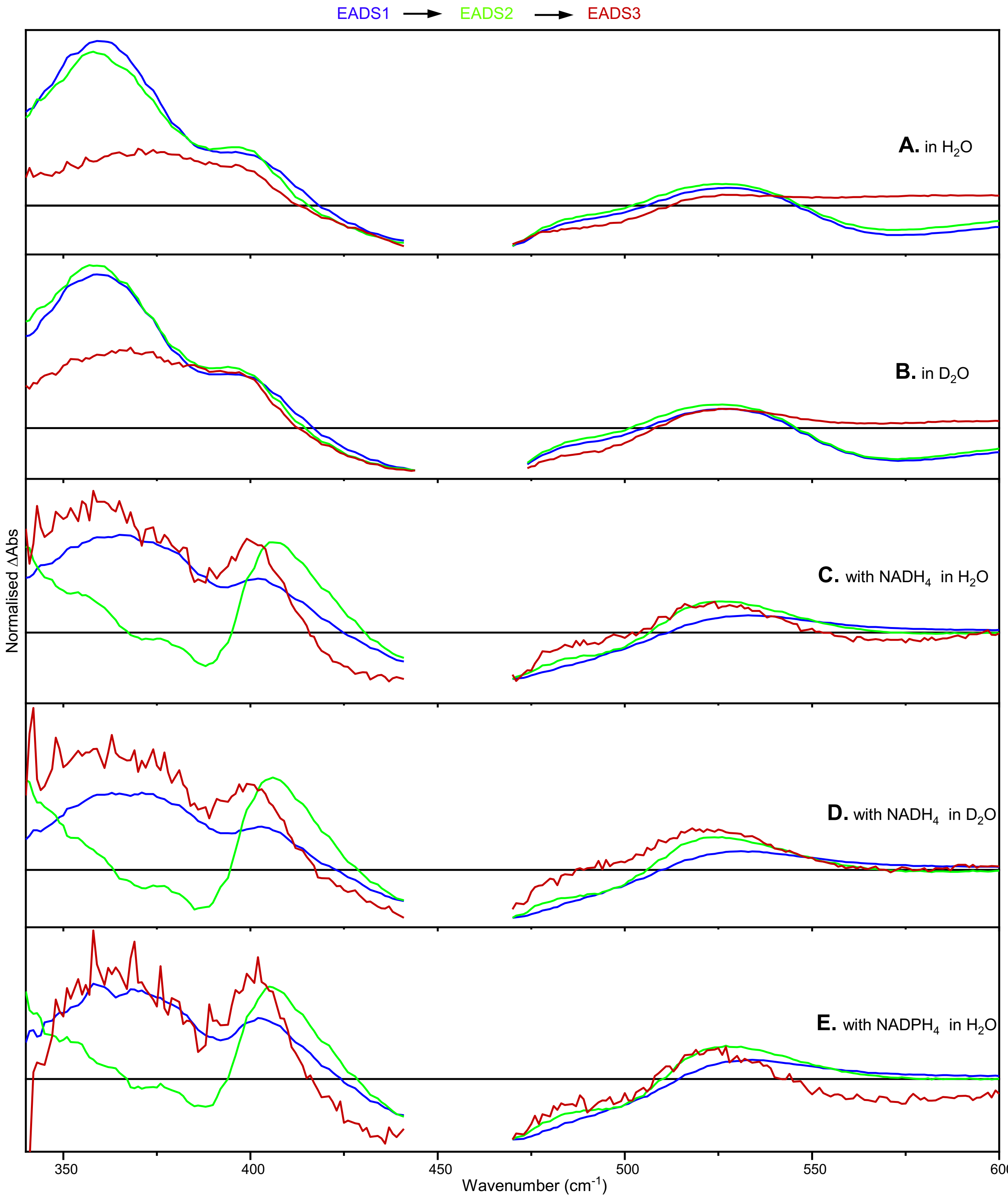

Figure S2. EADS resulting from global analysis of TRVis data using a sequential model of 3 inter-converting components normalised to the most intense negative feature. ^{15}N PETNR:L FMN in H_2O (A) and D_2O (B), ^{15}N PETNR:L FMN: $NADH_4$ in H_2O (C) and D_2O (D), and ^{15}N PETNR:L FMN: $NADPH_4$ in H_2O (E)

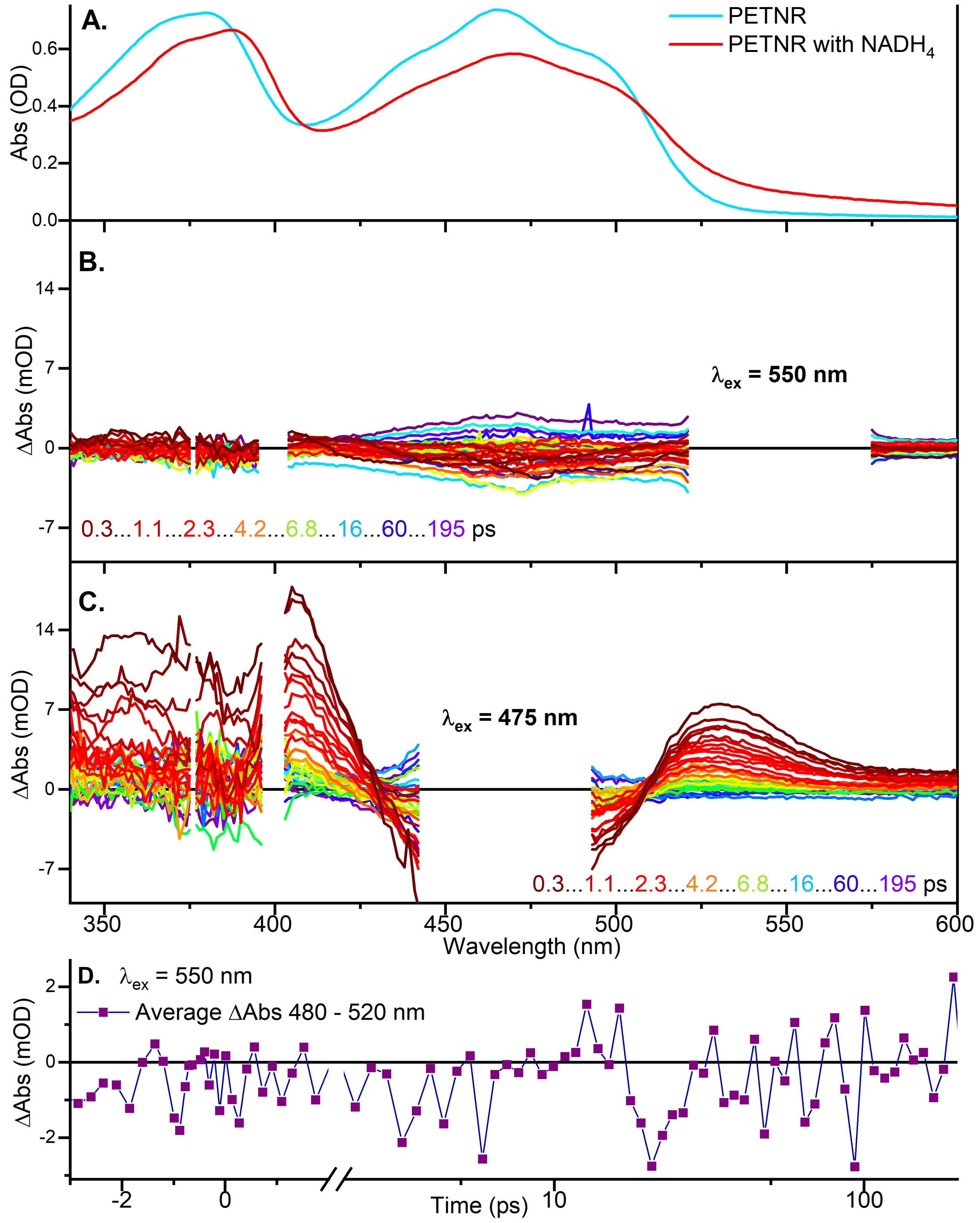

Figure S3. Measurements of $NADH_4$-bound L PETNR:L FMN in H_2O. (**A**)Visible absorbance spectra recorded on the samples used. TRVis data shown as difference spectra at selected time points after (**B**) excitation at 500 nm (0.5 μJ) , and (**C**) excitation at 475 nm (0.5 μJ). (**D**) Transient averaged over the 480-520 nm region for the sample excited at 550 nm.

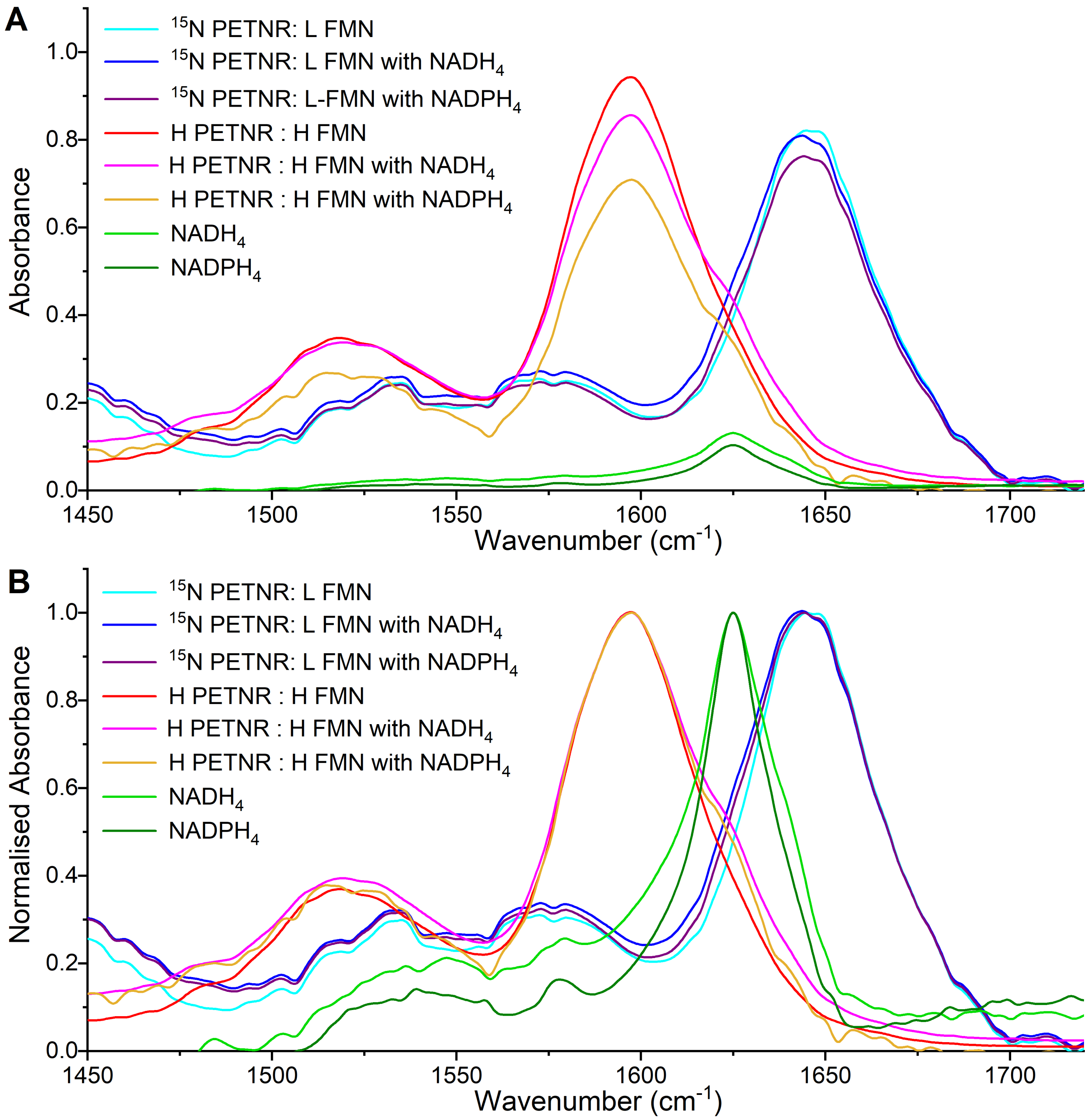

Figure S4. Infrared absorbance spectra recorded on the samples used in TRIR measurements (**A**) and normalised to maximum intensity feature (B). The spectra are dominated by the amide I and II bands at ca. 1600 – 1700 cm^{-1} and 1500 – 1600 cm^{-1} respectively in the ^{15}N-only labelled protein samples.

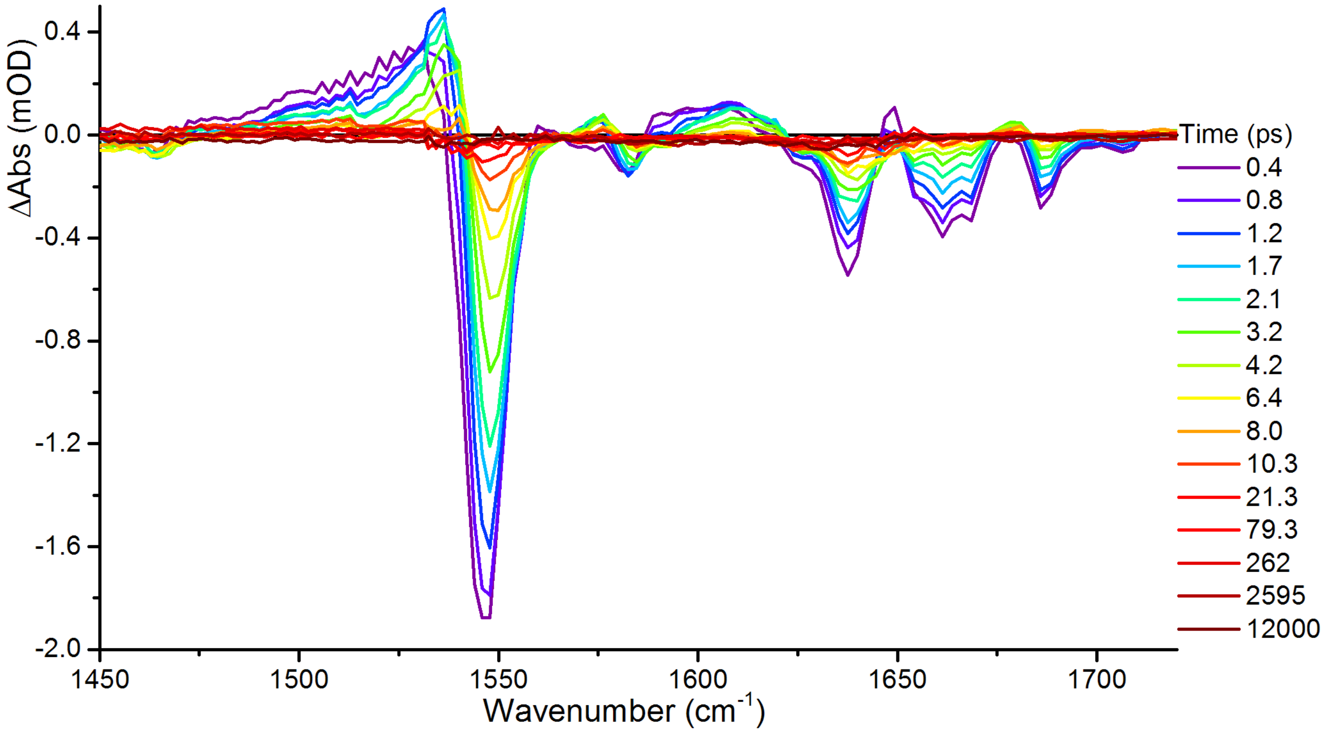

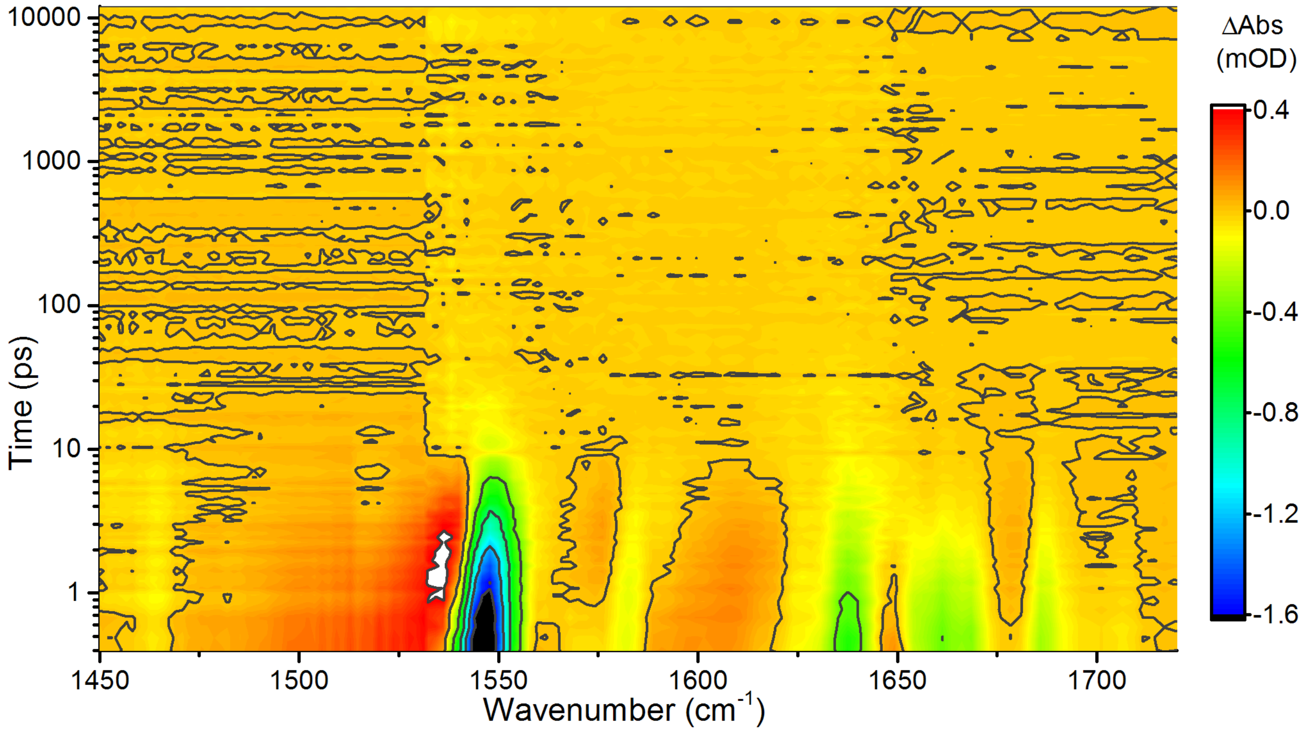

Figure S5. TRIR difference spectra at selected time points and contour plot of raw data for ^{15}N PETNR: L FMN : $NADH_4$

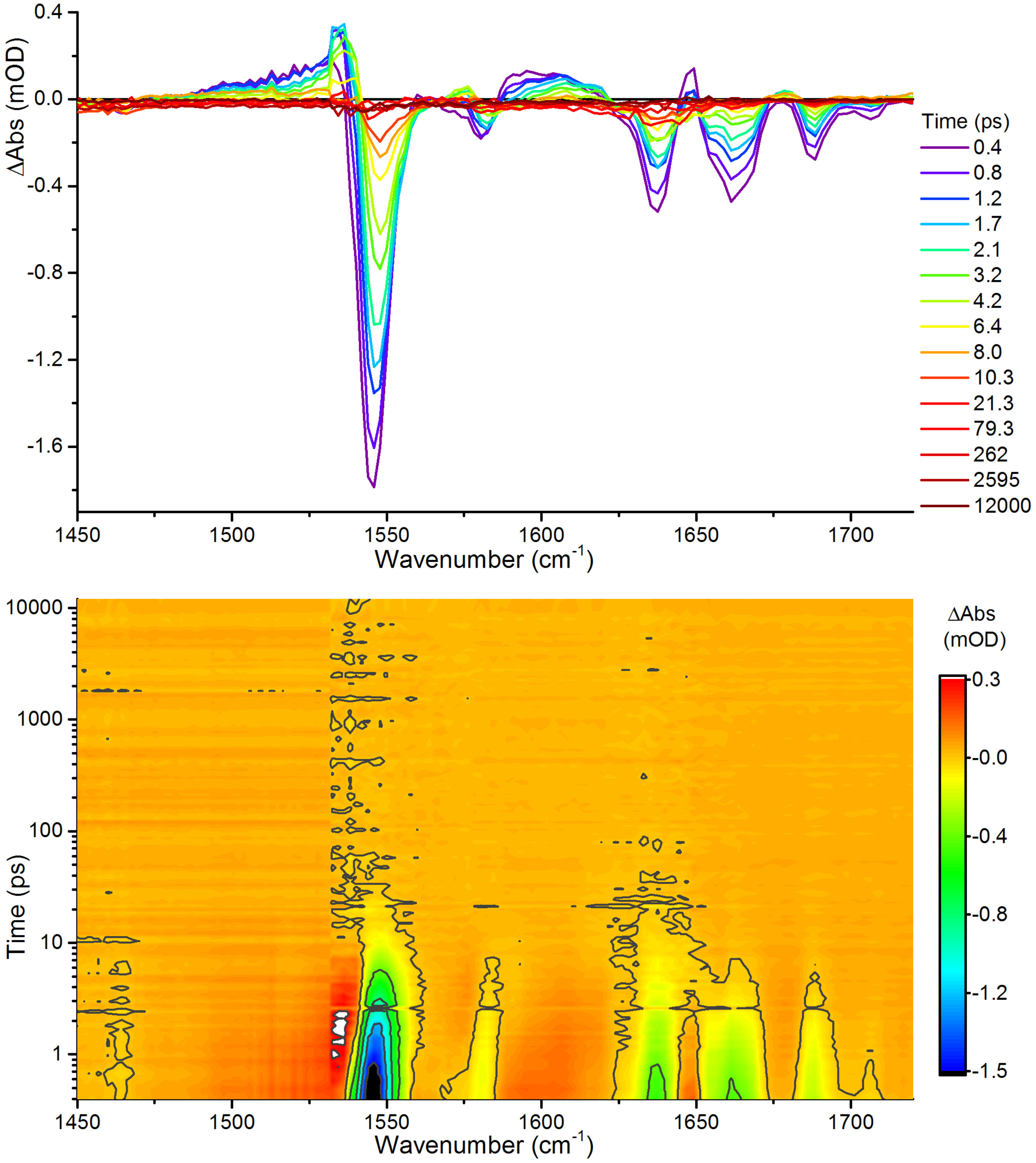

Figure S6. TRIR difference spectra at selected time points and contour plot of raw data for ^{15}N PETNR: L FMN : $NADPH_4$

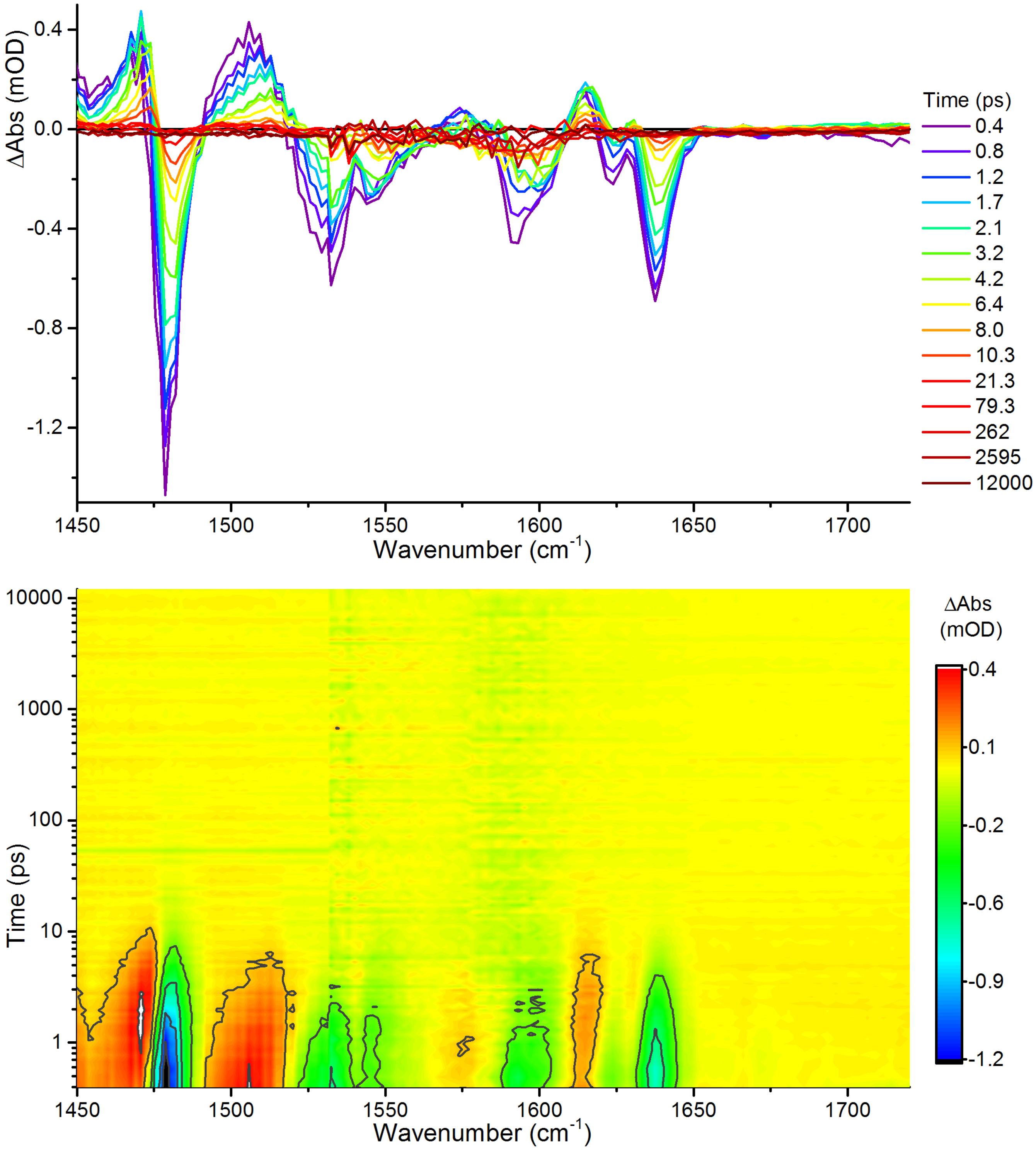

Figure S7. TRIR difference spectra at selected time points and contour plot of raw data for H PETNR: H FMN : $NADH_4$

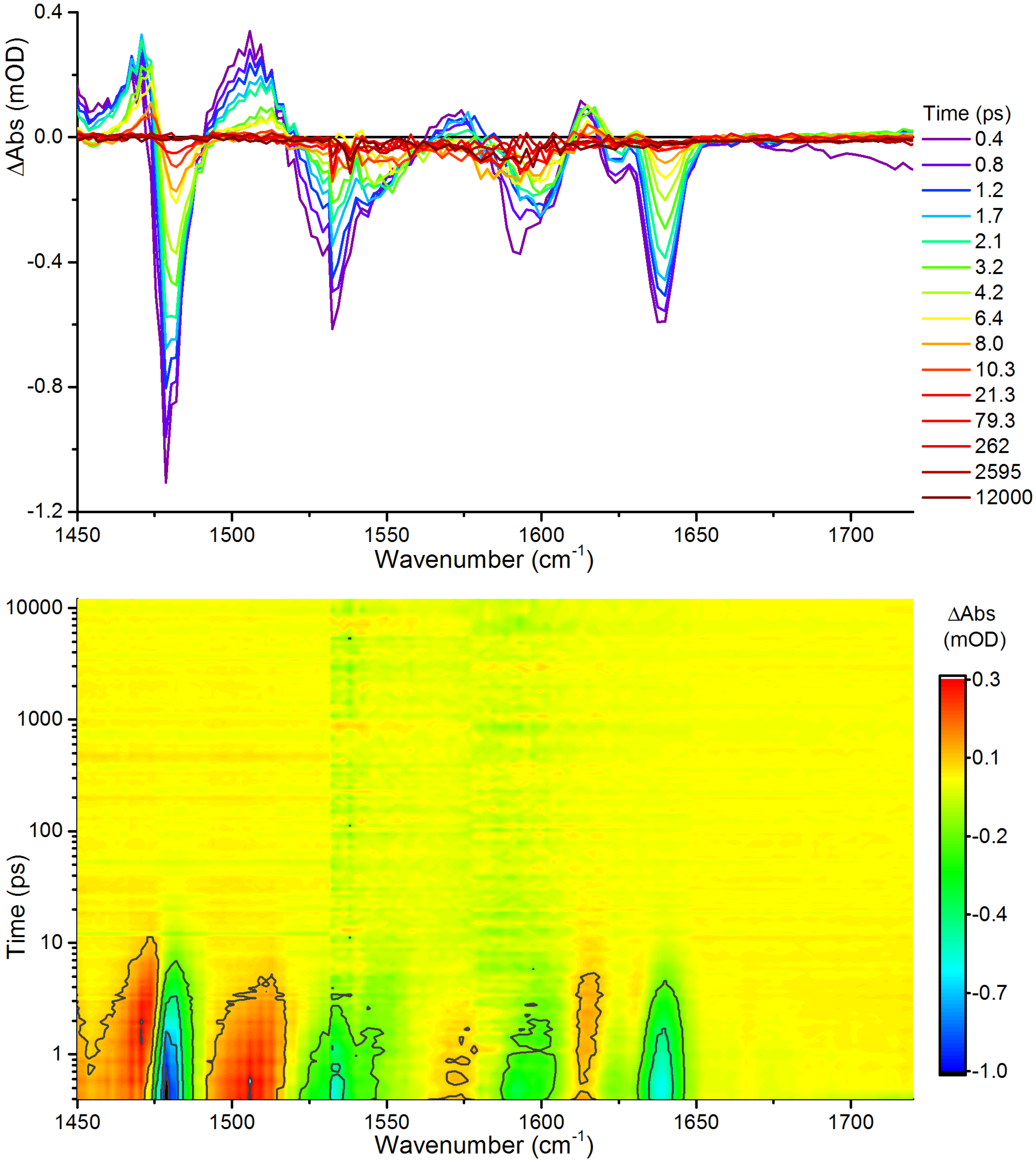

Figure S8. TRIR difference spectra at selected time points and contour plot of raw data for H PETNR: H FMN : $NADPH_4$

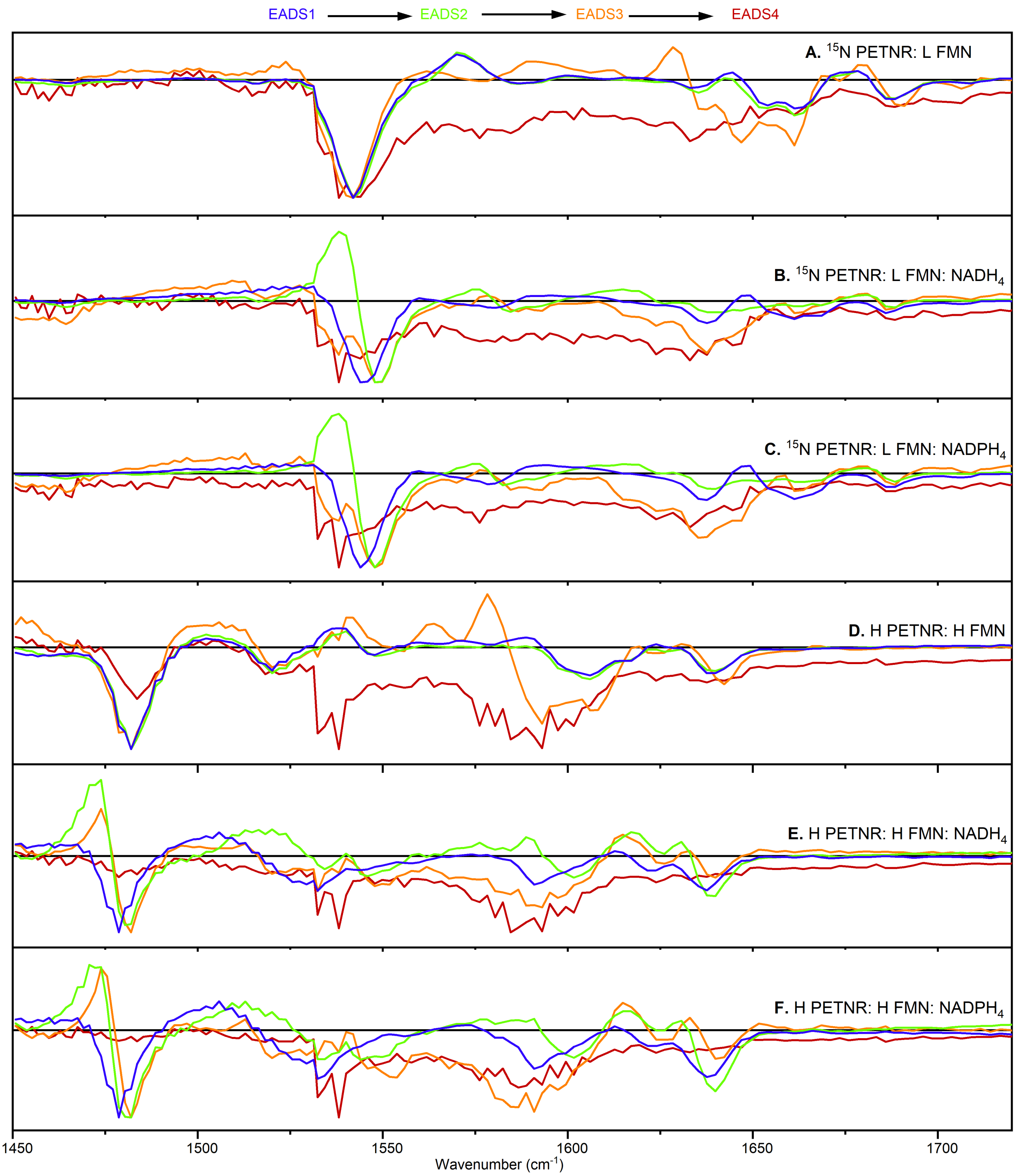

Figure S9. EADS resulting from global analysis of TRIR data using a sequential model of 4 inter-converting components normalized to the most intense negative feature. A. ^{15}N PETNR:L FMN (Data taken from Ref. [6]); B. ^{15}N PETNR:L FMN: $NADH_4$; C. ^{15}N PETNR:L FMN: $NADPH_4$; D. H PETNR:H FMN (Data taken from Ref. [6]);E. H PETNR:H FMN: $NADH_4$; F. H PETNR:H FMN: $NADPH_4$.

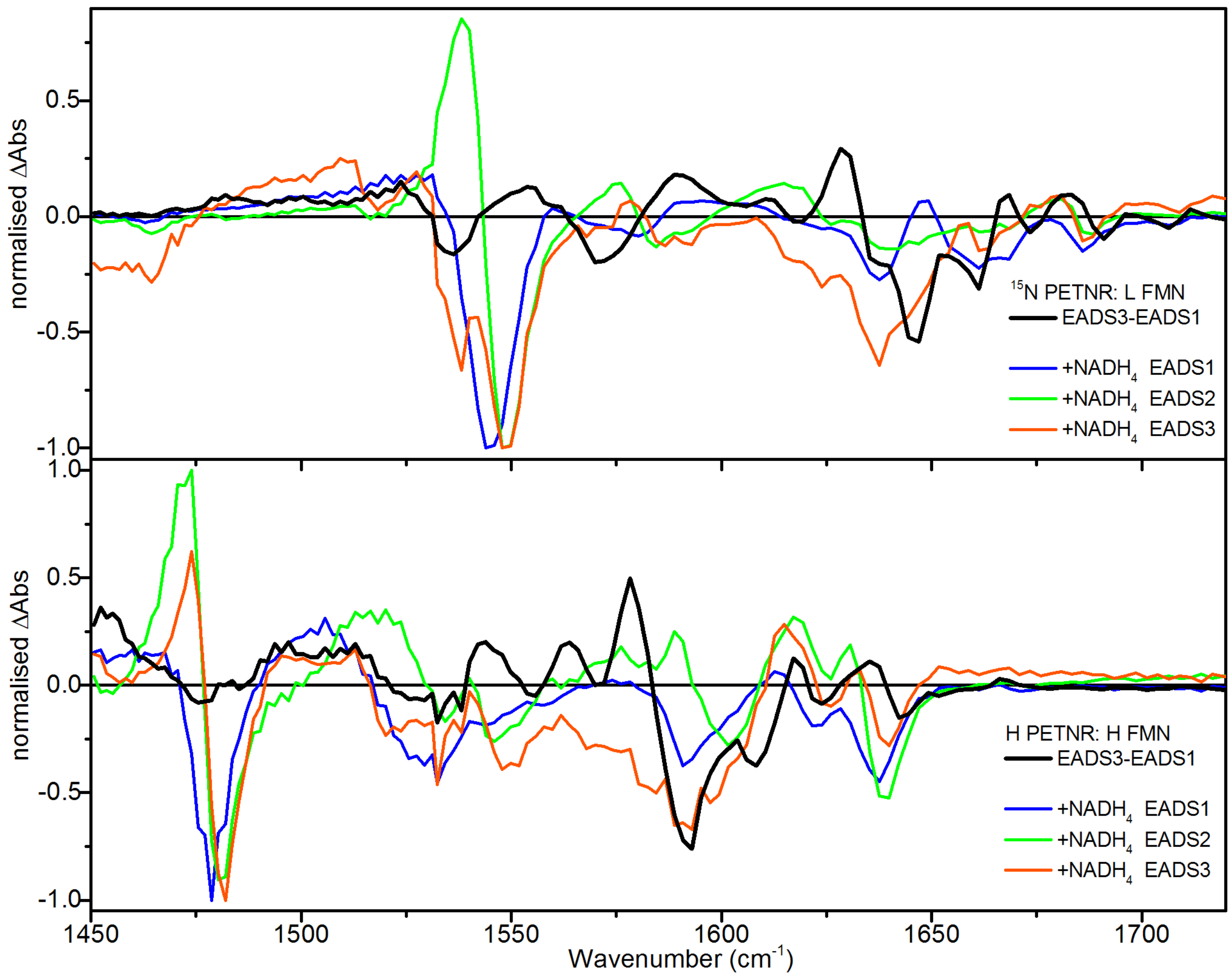

Figure S10. Normalized EADS3 with normalized EADS1 subtracted for PETNR samples (i.e. the features originating from the protein) overlaid with normalized EADS for cofactor bound samples of. ^{15}N PETNR:L FMN: $NADH_4$,. H PETNR:L FMN: $NADH_4$; and H PETNR:H FMN: $NADH_4$. Data without coenzyme analogue taken from Ref. [6]

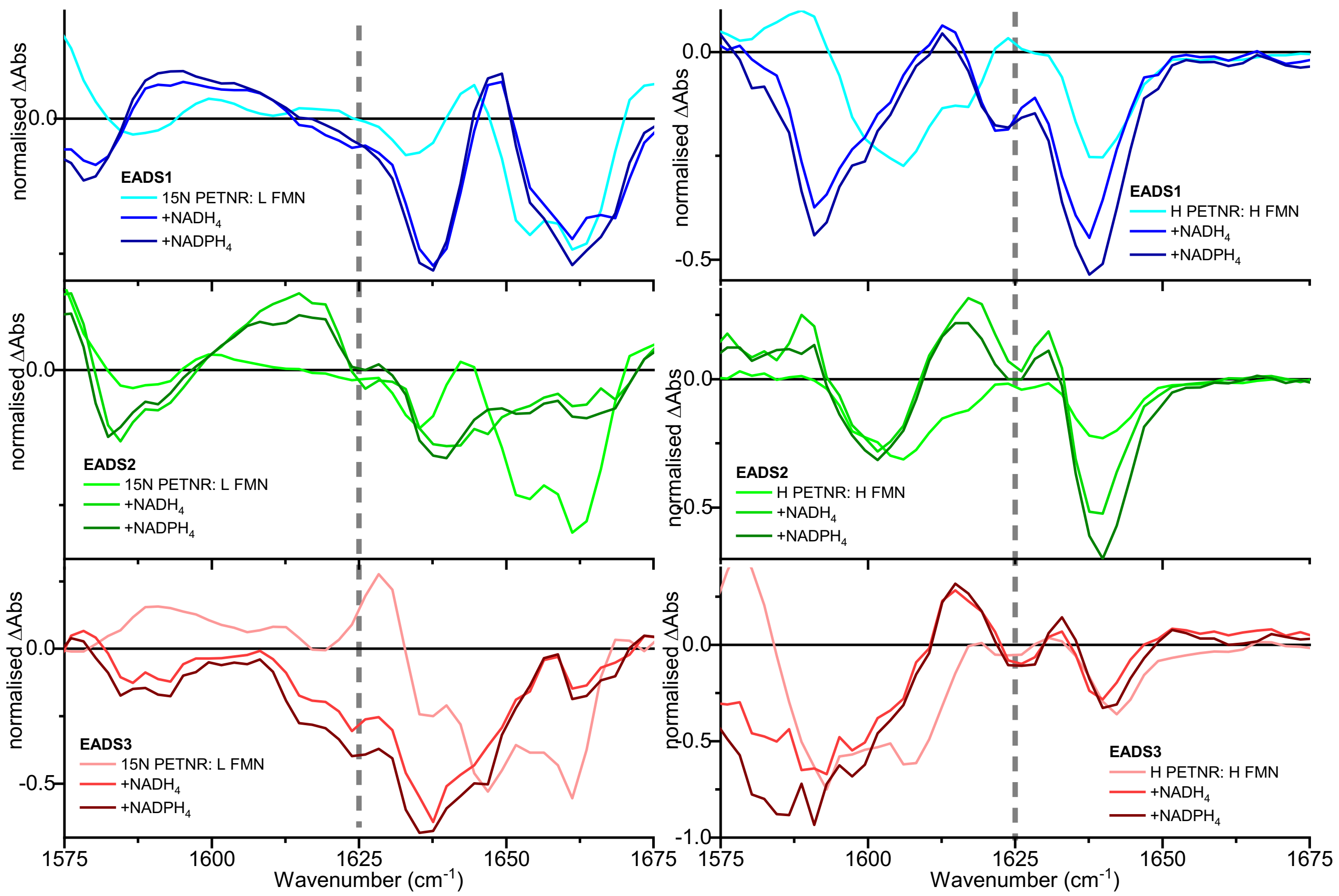

Figure S11. EADS resulting from global analysis of TRIR data using a sequential model of 4 inter-converting components normalized to the most intense negative feature, shown between 1575 and 1675 cm^{-1}. Dashed line indicates 1625 cm^{-1}, the approximate location of the C=O stretch in NAD(P)H_4 (see Figure S1). Only in EADS2 is there a clear negative feature at this position in both ^{15}N PETNR:L FMN and H PETNR:H FMN samples with coenzyme analogue, but not without. In the EADS1, there's no extra feature in the +NAD(P)H_4 spectra for the ^{15}N,L sample, which implies that the broad negative feature when the cofactors are added to the HH sample doesn't originate from NAD(P)H_4. In the EADS3, there may be a small feature at 1625 cm^{-1} in the ^{15}N,L sample, but it's not clear in the HH sample, which again makes its origin questionable. Data without coenzyme analogue taken from Ref. 6.

Additional computational data:

Table S1. (TD-)DFT cluster model energies

	Hartree-Fock energy	E(TD-HF/TD-DFT)
Model	Hartrees	Hartrees
Unoptimized crystal structure coordinates	-4587.417061	-
Geometry optimized ground state	-4587.99325348	-
Single point excited state	-4587.99325348	-4587.885785
Geometry optimized excited state	-	-4587.90946419

Table S2. DFT cluster model charge analysis. [a]

NBO electron occupancies	**(A)** DFT ground state	**(B)** TDDFT single point	**(C)** TD-DFT geometry optimized	**B – A**	**C – A**
NADH	177.9092	177.227	177.0180	**+0.682**	**+0.891**
FMN	198.0871	198.7644	198.8934	**-0.677**	**-0.806**
Thr26 + Leu25	77.98828	77.9958	78.0476	-0.008	-0.059
Gln100	47.96207	47.96654	47.9767	-0.004	-0.015
His181	52.03363	52.02847	52.0270	+0.005	+0.007
His184	52.04452	52.04231	52.0277	+0.002	+0.017
Asp324	63.97525	63.9755	64.0097	0.000	-0.034
Overall charge	670.0000	670.0000	670.0000	0.000	0.000

[a] Values computed using the Natural Bond Orbital (NBO) analysis.

Table S3. Cartesian coordinates for the geometry optimized (TD)DFT cluster models

		location	ground state	first singlet excited state
1	H	protein	1.29568, -6.46229, -0.98994	-1.25875, 6.51126, 0.92444
2	C	protein	1.03491, -5.58329, -0.40093	-0.79416, 5.57859, 1.24447
3	C	protein	1.11084, -5.93401, 1.06794	-1.0262, , 5.38497, 2.72605
4	O	protein	0.83542, -7.05417, 1.49954	-0.94699, 6.30917, 3.5351
5	H	protein	0.00477, -5.30048, -0.63878	0.28392, 5.65635, 1.07674
6	N	protein	1.44384, -4.92557, 1.90167	-1.27433, 4.11799, 3.12681
7	C	protein	1.44714, -5.1457, , 3.32803	-1.31694, 3.82553, 4.54332
8	C	protein	0.17406, -5.88741, 3.74472	-0.09901, 4.43381, 5.23773
9	O	protein	-0.92308, -5.46945, 3.45488	1.02159, 4.28237, 4.80827
10	C	protein	1.45733, -3.78072, 4.04643	-1.25557, 2.30151, 4.75749
11	O	protein	2.34371, -2.88084, 3.40321	-2.35696, 1.64061, 4.17369
12	C	protein	1.76565, -3.91818, 5.52784	-1.23971, 1.928, , 6.23052
13	C	protein	10.38225, -3.64975, -1.19172	-10.16317, 4.12503, -0.11391
14	C	protein	8.9842, -3.02831, -1.17802	-8.79835, 3.45179, -0.24896
15	C	protein	9.01087, -1.4959, -1.16665	-8.90644, 1.96467, -0.6009
16	C	protein	7.61332, -0.90472, -1.09259	-7.53798, 1.30904, -0.66217
17	O	protein	6.76602, -1.38717, -0.32823	-6.70507, 1.52152, 0.23024
18	N	protein	7.3457, , 0.14518, -1.88039	-7.27987, 0.51438, -1.70789
19	C	protein	10.03036, 2.38899, -0.37531	-9.9209, -1.9044, -1.0324
20	C	protein	9.0935, , 2.62133, 0.81348	-9.06481, -2.69979, -0.04286
21	C	protein	7.63717, 2.56618, 0.45209	-7.59733, -2.59975, -0.33492
22	N	protein	7.11623, 3.30258, -0.59186	-7.0901, -2.92051, -1.57773
23	C	protein	6.63249, 1.85879, 1.06466	-6.57657, -2.21763, 0.4977
24	C	protein	5.82511, 3.03851, -0.60039	-5.79043, -2.73198, -1.48914
25	N	protein	5.48052, 2.1717, , 0.38327	-5.42683, -2.31164, -0.25217
26	C	protein	3.26363, 6.7078, , 1.27847	-3.24556, -6.64248, -0.62514
27	C	protein	3.02872, 5.2082, , 1.06517	-2.90654, -5.15876, -0.43675
28	C	protein	2.83409, 4.85444, -0.37824	-2.87142, -4.40594, -1.73275
29	N	protein	2.47517, 3.59341, -0.80186	-2.5351, -3.07203, -1.8292
30	C	protein	2.95953, 5.58616, -1.53325	-3.15245, -4.78199, -3.02298
31	C	protein	2.40297, 3.61943, -2.15926	-2.6298, -2.71137, -3.13696
32	N	protein	2.6879, , 4.81036, -2.6409	-3.00086, -3.72193, -3.89297
33	C	protein	-6.20548, -5.57623, -4.67141	6.49139, 6.63801, -2.81245
34	C	protein	-6.14323, -4.04988, -4.77128	6.29983, 5.2999, -3.51973
35	C	protein	-6.75926, -3.3671, -3.55288	6.87768, 4.13454, -2.72219
36	C	protein	-6.62797, -1.84478, -3.54828	6.5622, , 2.78152, -3.35087
37	N	protein	-7.13344, -1.32304, -2.28792	7.03234, 1.70108, -2.5034
38	C	protein	-6.74075, -0.16935, -1.90233	6.29837, 0.65577, -2.41579
39	N	protein	-5.93908, 0.7145, -2.64594	5.12549, 0.42779, -3.14071
40	N	protein	-6.97121, 0.25052, -0.60513	6.54815, -0.30471, -1.45017
41	N	FMN	2.21537, 0.22849, -1.76054	-2.25412, 0.14191, -1.58646
42	C	FMN	3.56824, 0.12365, -1.51568	-3.58714, 0.25309, -1.32619
43	O	FMN	4.37546, 0.8422, -2.08895	-4.44124, -0.14628, -2.12389
44	N	FMN	4.03531, -0.83344, -0.60859	-3.98551, 0.84088, -0.13038
45	C	FMN	3.27195, -1.7693, , 0.03303	-3.13932, 1.42328, 0.78088
46	O	FMN	3.73057, -2.6461, , 0.74307	-3.58239, 1.99037, 1.79004
47	C	FMN	1.81072, -1.61493, -0.21326	-1.72836, 1.30155, 0.47811

48	N	FMN	1.00264, -2.39468, 0.42559	-0.84206, 1.82882, 1.35398
49	C	FMN	-0.33275, -2.29862, 0.17061	0.46048, 1.84184, 0.96199
50	C	FMN	-1.21959, -3.16389, 0.84877	1.40758, 2.46987, 1.79753
51	C	FMN	-2.56875, -3.15738, 0.57617	2.74381, 2.57723, 1.45361
52	C	FMN	-3.50896, -4.07405, 1.30829	3.70209, 3.27822, 2.38078
53	C	FMN	-3.05174, -2.27113, -0.42746	3.17185, 2.04207, 0.22006
54	C	FMN	-4.50594, -2.24157, -0.78228	4.5973, , 2.19685, -0.23138
55	C	FMN	-2.19566, -1.40035, -1.08172	2.25696, 1.37921, -0.60431
56	C	FMN	-0.83057, -1.37304, -0.77243	0.90833, 1.25674, -0.25923
57	N	FMN	0.04992, -0.47431, -1.35998	-0.03754, 0.58472, -1.05887
58	C	FMN	1.38886, -0.58478, -1.14323	-1.37727, 0.67157, -0.7312
59	C	FMN	-0.47047, 0.70837, -2.07808	0.34172, -0.01784, -2.34908
60	C	FMN	-0.44521, 0.63109, -3.61055	0.08291, 0.91971, -3.54671
61	O	FMN	0.84604, 0.9129, -4.11638	-1.25039, 0.82912, -4.00554
62	C	FMN	-1.39334, 1.70215, -4.19693	0.94536, 0.6164, -4.77506
63	O	FMN	-1.39371, 2.87609, -3.4036	0.77079, -0.70766, -5.25239
64	C	FMN	-2.81256, 1.19159, -4.50262	2.44344, 0.90584, -4.6278
65	O	FMN	-3.23719, 0.17245, -3.60183	2.67956, 2.03818, -3.80588
66	C	FMN	-2.8986, , 0.61461, -5.91452	3.0722, , 1.15289, -6.00485
67	O	FMN	-4.1942, , 0.12634, -6.19891	4.40218, 1.63695, -5.8984
68	O	NADH4	-5.1564, , 2.69334, 3.42869	5.21348, -3.62858, 2.59132
69	C	NADH4	-4.90225, 3.90177, 2.69658	4.96643, -4.63242, 1.59702
70	C	NADH4	-4.13864, 3.55559, 1.4356	4.15921, -4.01412, 0.47798
71	O	NADH4	-2.87395, 3.03472, 1.79729	2.91172, -3.58743, 1.01656
72	C	NADH4	-4.77031, 2.46135, 0.55879	4.77411, -2.77156, -0.19391
73	O	NADH4	-5.65913, 2.95714, -0.42556	5.38269, -3.07756, -1.43058
74	C	NADH4	-3.55172, 1.83886, -0.15468	3.55673, -1.84711, -0.48293
75	O	NADH4	-3.4204, , 2.33085, -1.47737	3.31443, -1.64852, -1.85194
76	C	NADH4	-2.33738, 2.28975, 0.70936	2.37355, -2.58995, 0.19787
77	N	NADH4	-1.53021, 1.22823, 1.23716	1.56091, -1.76222, 1.09742
78	C	NADH4	-0.17063, 1.33056, 1.26394	0.24829, -1.81142, 1.03787
79	C	NADH4	0.64236, 0.55051, 2.02207	-0.57598, -1.23923, 2.02826
80	C	NADH4	2.09462, 0.68072, 1.84149	-2.05279, -1.3583, , 1.8114
81	O	NADH4	2.62502, 1.50282, 1.05908	-2.49121, -1.9477, , 0.81327
82	N	NADH4	2.86648, -0.14955, 2.56907	-2.82779, -0.83083, 2.76045
83	C	NADH4	0.06381, -0.37631, 3.06521	0.0216, -0.68978, 3.27093
84	C	NADH4	-1.37091, 0.0439, , 3.39186	1.44956, -1.19501, 3.48117
85	C	NADH4	-2.18008, 0.24778, 2.1157	2.26227, -1.07655, 2.201
86	O	NADH4	-7.38114, 3.17671, 4.37825	7.44967, -4.22678, 3.45216
87	P	NADH4	-5.89958, 2.81885, 4.84175	5.9416, -4.09159, 3.94363
88	O	NADH4	-6.01682, 1.29301, 5.27412	5.95899, -2.73557, 4.77317
89	O	NADH4	-5.27991, 3.7338, , 5.817	5.35855, -5.26997, 4.60903
90	H	protein	1.69267, -4.75074, -0.65671	-1.17985, 4.74499, 0.65347
91	H	protein	1.69057, -4.00138, 1.54707	-1.24005, 3.34359, 2.45379
92	H	protein	2.31885, -5.74204, 3.63304	-2.22939, 4.22682, 5.00212
93	H	protein	0.30117, -6.78416, 4.37537	-0.28221, 4.96126, 6.19115
94	H	protein	0.46369, -3.33808, 3.90641	-0.31149, 1.97605, 4.29167
95	H	protein	3.23266, -3.26102, 3.35861	-2.5679, , 1.97046, 3.2723
96	H	protein	1.72938, -2.93645, 6.00393	-1.24851, 0.83986, 6.32529
97	H	protein	2.76294, -4.34395, 5.67432	-2.1258, , 2.32636, 6.73215

98	H	protein	1.03854, -4.5695, , 6.02245	-0.34496, 2.30635, 6.73117
99	H	protein	10.94285, -3.3377, -2.07811	-10.72855, 4.0582, -1.04837
100	H	protein	10.32261, -4.74062, -1.19629	-10.05495, 5.18215, 0.14007
101	H	protein	10.95393, -3.34489, -0.3097	-10.75758, 3.64723, 0.671
102	H	protein	8.42696, -3.36571, -0.29934	-8.23473, 3.54146, 0.68402
103	H	protein	8.42035, -3.36685, -2.05481	-8.20902, 3.95882, -1.02151
104	H	protein	9.54391, -1.10945, -2.0418	-9.44422, 1.82733, -1.54492
105	H	protein	9.55286, -1.14478, -0.27849	-9.48013, 1.44581, 0.17774
106	H	protein	6.43098, 0.59165, -1.8317	-6.36066, 0.07685, -1.79686
107	H	protein	8.0527, , 0.55051, -2.47253	-7.97773, 0.33672, -2.41232
108	H	protein	9.86663, 1.39666, -0.80724	-9.69754, -0.83609, -0.9607
109	H	protein	11.0773, 2.46261, -0.07086	-10.9868, -2.04694, -0.83754
110	H	protein	9.84375, 3.12865, -1.15781	-9.70858, -2.22312, -2.05596
111	H	protein	9.31895, 3.60094, 1.25405	-9.36903, -3.75334, -0.07653
112	H	protein	9.28716, 1.87939, 1.5955	-9.24882, -2.35473, 0.97973
113	H	protein	6.63956, 1.17745, 1.90124	-6.56768, -1.89706, 1.52779
114	H	protein	5.09531, 3.43077, -1.29703	-5.07118, -2.86634, -2.28557
115	H	protein	4.53748, 1.84076, 0.59225	-4.48374, -2.09539, 0.05919
116	H	protein	4.15993, 7.03919, 0.7468	-4.22896, -6.7586, -1.08879
117	H	protein	3.39626, 6.92716, 2.33949	-3.25838, -7.15571, 0.33794
118	H	protein	2.41508, 7.2893, , 0.90806	-2.50775, -7.13105, -1.26715
119	H	protein	2.15083, 4.88411, 1.6371	-1.93781, -5.05852, 0.0676
120	H	protein	3.87817, 4.63819, 1.46405	-3.6431, -4.69128, 0.22915
121	H	protein	2.3525, , 2.77529, -0.20013	-2.31392, -2.44989, -1.05705
122	H	protein	2.12762, 2.74578, -2.73556	-2.42001, -1.70144, -3.46272
123	H	protein	-5.66162, -5.92708, -3.78876	5.99646, 6.63314, -1.83629
124	H	protein	-5.76687, -6.05415, -5.55107	6.07846, 7.46544, -3.39527
125	H	protein	-7.24142, -5.91814, -4.58218	7.55368, 6.84197, -2.64478
126	H	protein	-6.65672, -3.71468, -5.68112	6.7639, , 5.33079, -4.51338
127	H	protein	-5.09723, -3.73202, -4.87257	5.22872, 5.12388, -3.68524
128	H	protein	-6.27903, -3.74897, -2.64338	6.4599, , 4.1445, -1.707
129	H	protein	-7.82163, -3.62974, -3.47218	7.96352, 4.24707, -2.61497
130	H	protein	-5.56523, -1.57649, -3.6971	5.4716, , 2.73667, -3.51474
131	H	protein	-7.17541, -1.42568, -4.40864	7.02153, 2.7228, -4.34994
132	H	protein	-6.19295, 0.73285, -3.62899	5.19385, 0.61986, -4.13884
133	H	protein	-5.9232, , 1.65399, -2.25859	4.67792, -0.45807, -2.92664
134	H	protein	-7.11886, 1.24296, -0.4722	6.38161, -1.26853, -1.72184
135	H	protein	-7.63474, -0.33733, -0.11972	7.43235, -0.1591, -0.98128
136	H	FMN	5.0641, -0.92324, -0.49805	-4.99875, 0.98413, 0.01744
137	H	FMN	-0.80593, -3.84174, 1.59219	1.05266, 2.92085, 2.7216
138	H	FMN	-4.32089, -3.50883, 1.77667	4.56037, 2.6428, , 2.6195
139	H	FMN	-2.97524, -4.62889, 2.08193	3.19942, 3.55123, 3.31054
140	H	FMN	-3.97039, -4.79198, 0.62229	4.09554, 4.19328, 1.92605
141	H	FMN	-4.66013, -1.6726, -1.69972	4.6543, , 2.21484, -1.32108
142	H	FMN	-5.09453, -1.761, , 0.00911	5.22786, 1.36611, 0.10727
143	H	FMN	-4.90991, -3.2479, -0.92101	5.0374, , 3.12255, 0.14596
144	H	FMN	-2.58663, -0.77338, -1.8743	2.62002, 0.98746, -1.54773
145	H	FMN	0.1404, , 1.56165, -1.77141	-0.24696, -0.9312, -2.47359
146	H	FMN	-1.48788, 0.86605, -1.72145	1.38987, -0.31373, -2.27906
147	H	FMN	-0.77946, -0.37059, -3.92297	0.32542, 1.94231, -3.2213

148	H	FMN	1.50613, 0.64117, -3.45	-1.80699, 0.59141, -3.23231
149	H	FMN	-0.93547, 1.99482, -5.14995	0.53218, 1.25782, -5.56335
150	H	FMN	-2.02253, 2.74858, -2.674	1.18899, -1.31753, -4.6299
151	H	FMN	-3.49249, 2.05187, -4.43794	2.90295, 0.01905, -4.16547
152	H	FMN	-4.00841, 0.48212, -3.08891	3.49392, 1.84131, -3.31228
153	H	FMN	-2.67881, 1.39635, -6.64685	3.10086, 0.22513, -6.58029
154	H	FMN	-2.14139, -0.17793, -6.0255	2.45154, 1.87787, -6.54874
155	H	FMN	-4.38635, -0.51151, -5.49669	4.33877, 2.50154, -5.46889
156	H	NADH4	-4.30803, 4.58473, 3.31029	4.40976, -5.46161, 2.04229
157	H	NADH4	-5.85242, 4.38478, 2.4416	5.91759, -5.00623, 1.20274
158	H	NADH4	-4.03035, 4.4747, , 0.83777	3.99404, -4.77753, -0.29494
159	H	NADH4	-5.25844, 1.70857, 1.18684	5.47175, -2.26736, 0.48297
160	H	NADH4	-6.2664, , 3.60293, -0.04087	6.11517, -3.69226, -1.2885
161	H	NADH4	-3.62572, 0.74661, -0.20229	3.70607, -0.85678, -0.04444
162	H	NADH4	-4.11349, 2.99804, -1.61445	3.56709, -2.45697, -2.32549
163	H	NADH4	-1.69967, 2.92603, 0.08159	1.7071, -2.99075, -0.57523
164	H	NADH4	0.25681, 2.0793, , 0.59956	-0.2025, -2.35559, 0.21384
165	H	NADH4	2.49892, -0.98812, 3.01133	-2.48858, -0.15148, 3.44339
166	H	NADH4	3.85977, -0.12854, 2.38645	-3.8261, -0.84804, 2.59833
167	H	NADH4	0.0895, -1.42132, 2.72645	0.01094, 0.4069, , 3.17365
168	H	NADH4	-3.17794, 0.62391, 2.35663	3.23386, -1.56109, 2.30846
169	H	protein	3.22679, 6.63006, -1.6243	-3.45764, -5.7596, -3.36974
170	H	NADH4	-1.86237, -0.71105, 4.01016	1.94093, -0.61494, 4.26297
171	H	NADH4	-2.28489, -0.70242, 1.58077	2.40434, -0.03118, 1.90416
172	H	NADH4	0.6689, -0.3322, , 3.97828	-0.60833, -0.93407, 4.13108
173	H	NADH4	-7.90632, 3.60096, 5.07206	7.98821, -4.79649, 4.02041
174	H	protein	-1.35881, 0.9848, , 3.9525	1.43127, -2.24283, 3.79443
175	H	protein	-5.28805, 1.00268, 5.84164	5.18287, -2.62796, 5.3423

Additional references:

(1) Iorgu, A. I.; Cliff, M. J.; Waltho, J. P.; Scrutton, N. S.; Hay, S., Chapter six - Isotopically labeled flavoenzymes and their uses in probing reaction mechanisms. In *Methods Enzymol.*, Palfey, B. A., Ed. Academic Press: **2019**; *620*, 145-166.

(2) Longbotham, J. E.; Hardman, S. J. O.; Görlich, S.; Scrutton, N. S.; Hay, S., Untangling heavy protein and cofactor isotope effects on enzyme-catalyzed hydride transfer. *J. Am. Chem. Soc.* **2016,** *138*, 13693-13699.

(3) Iorgu, A. I.; Baxter, N. J.; Cliff, M. J.; Waltho, J. P.; Hay, S.; Scrutton, N. S., 1H, 15N and 13C backbone resonance assignments of pentaerythritol tetranitrate reductase from Enterobacter cloacae PB2. *Biomol. NMR Assign.* **2018,** *12*, 79-83.

(4) Pudney, C. R.; Hay, S.; Scrutton, N. S., Practical aspects on the use of kinetic isotope effects as probes of flavoprotein enzyme mechanisms. In *Flavins and flavoproteins: Methods and protocols*, Weber, S., Schleicher, E. Eds.; Springer New York, **2014**, 161-175.

(5) Snellenburg, J. J.; Laptenok, S. P.; Seger, R.; Mullen, K. M.; van Stokkum, I. H. M., Glotaran: a Java-based graphical user interface for the R package TIMP. *J. Stat. Softw.* **2012,** *49*, 1-22.

(6) Hardman, S. J. O.; Iorgu, A. I.; Heyes, D. J.; Scrutton, N. S.; Sazanovich, I. V.; Hay, S., Ultrafast vibrational energy transfer between protein and cofactor in a flavoenzyme. *J. Phys. Chem. B* **2020,** *124*, 5163-5168.
