# Supplementary material for: Photoinduced Electron Transfer from a 1,4,5,6-Tetrahydro Nicotinamide Adenine Dinucleotide (Phosphate) Analogue to Oxidized Flavin in an Ene-Reductase Flavoenzyme
Source: J Phys Chem Lett. 2023 Mar 27;14(13):3236–42. doi: 10.1021/acs.jpclett.3c00176 (PMC10084465; doi:10.1021/acs.jpclett.3c00176)
Supplement: Supplementary file 2 — jz3c00176_si_002.pdf [file jz3c00176_si_002.pdf]

jz-2023-001767.R1

Name: Peer Review Information for "Photoinduced Electron Transfer from a 1,4,5,6-Tetrahydro Nicotinamide Adenine Dinucleotide (Phosphate) Analog to Oxidized Flavin in an Ene-Reductase Flavoenzyme"

First Round of Reviewer Comments

Reviewer: 1

Comments to the Author

This is an interesting paper, and I fully support its publication, but I have both a suggestion and a question. The suggestion is the paper needs to state up front what the point actually may be. Statements early in the paper suggest that the oxidized form of the flavin may be a better photo-catalyst, because of greater ability to absorb light, but then concerns are raised about the back reaction (the electron transferring back to the flavin.) Unless I reads the paper absolutely incorrectly, that is exactly what the investigator found. Thus the investigator have to state at the outset either that they will be proposing new ways around this as supported by the highly technical work in the manuscript, or they have to state that existing concerns regarding the use of oxidized flavins were exactly correct and the point to the manuscript is to warn away other investigator.

My question regards the DFT results. As a non-expert the fact that the ground state and the excited state structures seem almost superimposable is surprising. Should not one expect some geometric changes - the excited state structure is said to be geometry optimized. A comment about this is in order.

Reviewer: 2

Comments to the Author

1. What is the major advance reported in the paper?

The authors propose to use photoexcited oxidized flavoenzymes to catalyze non-natural photochemical reactions, which is supposed to have certain practical advantages over their reduced analogues.

The authors show that it is possible to generate a short lived FMN semiquinone upon VIS excitation in a flavoenzyme. However, - as they claim - it is challenging to use this oxidized ene-reductase enzyme(s) because of a rapid back ET to the ground state.

2. What is the immediate significance of this advance?

The authors authors show that the envisaged catalytic pathway (advantages mentioned above) is not feasible and a successful implementation will require alternative strategies, some of which the authors briefly mention in the conclusions.

3. Technical suggestions

- What are the fluences of the excitation beams (mJ/cm^2)?

- I would not call the change in the absorption spectra (upon addition of NADH) a "shift". Rather a new broad band emerges under the only slightly perturbed original absorption bands.

- Have the authors tried to explicitly excite the complex (beyond 550nm)? In their current experiment they excite into both the broad complex band and the "narrow" S1 band. If the broad band indeed represents a CT band, then partially exciting it would explain the immediate appearance of the CT state (and the lack of stimulated emission). Having a tunable light source for excitation, it might have been enlightening.

- Have the authors tried to estimate the involved free energy changes using the Weller equation and doing so rationalize the correspondingly fast rates for ET and BET?

- This observation "Below 400 nm there is a large positive absorption feature, which does not change in shape until the end of the measurement time frame." Is not correct, as would be easily visible from normalized EADS (EADS3 is different in the range < 400nm)

- It is a pity the authors have not performed emission (ss and tr) experiments, to better understand the nature of the different EADS (in the absence of NADH).

- The assignment of the first lifetime to ET is erroneous in my opinion, as the EADS already completely resembles that of the ionic state (complete lack of stimulated emission). Thus ET has to be faster than the lifetime of EADS1! Also, how is the ET assigned from the "different" structural conformation (EADS2 without NADH)? Does this subpopulation also undergo ET?

- Unfortunately the disentangling of the kinetics (both VIS and IR) is quite hand-wavy and thus remains pretty tentative. E.g. EADS1 in the presence of NADH seems to contain a mixture of features of the oxidized FMN (educt) and the semiquinone (product). If the trivial global fitting approach does not yield meaningful results, the authors should consider alternative means of analyzing their broadband spectra (see e.g. 10.1080/0144235X.2020.1757942).

Author's Response to Peer Review Comments:

Dear Professor Editor,

We thank you for the opportunity to submit a revised version of our manuscript.

The attached response to reviewer's comments describes the changes made to out manuscript.

Regards,

Sam Hay

Speirs *et al.* Response to reviewer's comments.

We thank the two reviewers for their comments, which we address point-by-point below. The non-scientific changes requested are also addressed at the end of this document.
We have included a copy of the manuscript with changes highlighted in yellow as an aid. Changes to the SI include an additional panel for Figure S1 and a new Figure S3, with other subsequent figures renumbered.

Reviewer 1:
This is an interesting paper, and I fully support its publication, but I have both a suggestion and a question. The suggestion is the paper needs to state up front what the point actually may be. Statements early in the paper suggest that the oxidized form of the flavin may be a better photo-catalyst, because of greater ability to absorb light, but then concerns are raised about the back reaction (the electron transferring back to the flavin.) Unless I reads the paper absolutely incorrectly, that is exactly what the investigator found. Thus the investigator have to state at the outset either that they will be proposing new ways around this as supported by the highly technical work in the manuscript, or they have to state that existing concerns regarding the use of oxidized flavins were exactly correct and the point to the manuscript is to warn away other investigator.

The reviewer has understood the manuscript correctly and we thank them for their suggestions. We make some suggestions in the final paragraph how one might deal with the challenges of using oxidised flavins in such applications, but agree that a more explicit warning of these challenges is appropriate. We have expanded the final paragraphs and have added: "*These [TRIR] experiments may be a useful tool for the enzyme engineer when optimizing or troubleshooting the de-sign of new flavoenzyme catalyzed photochemical reactions….While charge separation (forward eT) is favorable with appropriate substrates that can bind in close proximity to the flavin (i.e. with short eT distance and rapid eT that can out-compete reduction quenching), rapid relaxation of the charge separated state(s) via back eT will lead to non-productive charge recombination….Our work suggests that selecting slow back eT may be more important than optimizing charge separation, so any screening strategy should account for this.".*

My question regards the DFT results. As a non-expert the fact that the ground state and the excited state structures seem almost superimposable is surprising. Should not one expect some geometric changes - the excited state structure is said to be geometry optimized. A comment about this is in order.

The DFT model was geometry optimised in both the ground state and the first excited state. It appears that the enzyme active site preorganises and constrains the geometry of the FMN-NAD(P)H_4 complex such that there is little conformational change possible upon excitation to the S1 state. We have expanded our discussion of this point on p.5 to say: *"Comparison of the. S_0 and S_1 geometries show only minor changes in geometry, consistent with the geometrically constrained and preorganised (charge transfer) nature of the active site in the ground state."*

Reviewer 2:
1. What is the major advance reported in the paper? The authors propose to use photoexcited oxidized flavoenzymes to catalyze non-natural photochemical reactions, which is supposed to have certain practical advantages over their reduced analogues. The authors show that it is possible to generate a short lived FMN semiquinone upon VIS excitation in a flavoenzyme. However, - as they claim - it is challenging to use this oxidized ene-reductase enzyme(s) because of a rapid back ET to the ground state.
2. What is the immediate significance of this advance? The authors show that the envisaged catalytic pathway (advantages mentioned above) is not feasible and a successful

implementation will require alternative strategies, some of which the authors briefly mention in the conclusions.

Reviewer 1 also raised similar points, which we have addressed above with new text on p.5. Our work shows that charge separation is readily achieved using oxidized ene-reductase enzymes with appropriate substrates, so one should focus on reaction design that disfavours non-productive charge recombination. We hope that our changes have made this more clear.

3. Technical suggestions
- What are the fluences of the excitation beams (mJ/cm^2)?

We do not routinely measuring the bean diameter, so have not reported the fluence. Instead, we report the pulse energy (in the supporting information). The beam diameters are expected to be on the order of 50-250 µm, making the fluences around 1-20 mJ/cm^2 for the TRVis and 2-40 mJ/cm^2 for the TRIR measurements.

- I would not call the change in the absorption spectra (upon addition of NADH) a "shift". Rather a new broad band emerges under the only slightly perturbed original absorption bands.

The reviewer is correct, as a simple fitting of Gaussian functions to the absorption spectra shows (we have added this to Figure S1):

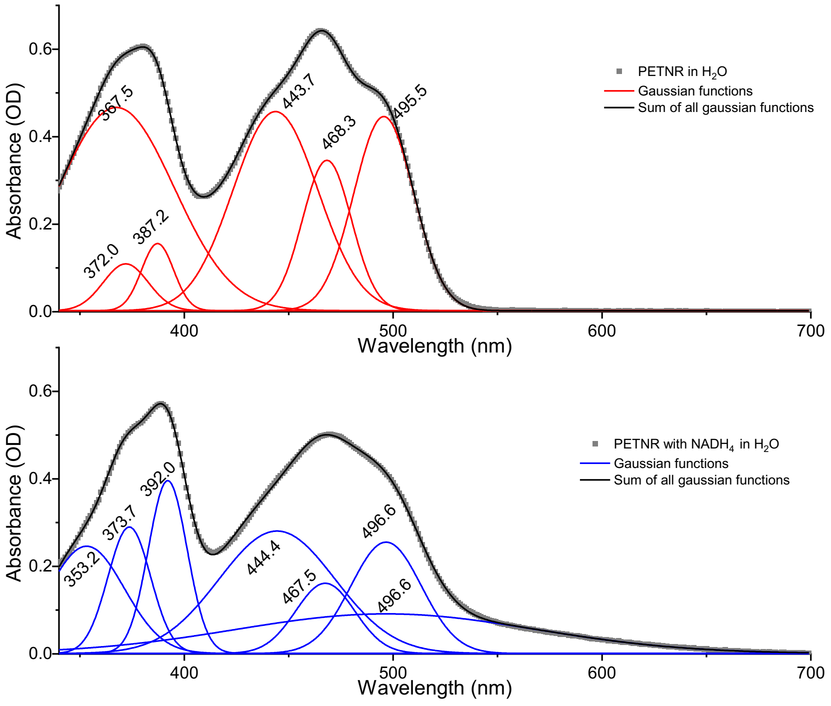

We have changed the offending sentence in manuscript on p.3 from:
"When NAD(P)H4 is bound, there are shifts in the ground state absorption spectra…" to *"…, there is an addition broad feature in the ground state absorption spectra features…".*

- Have the authors tried to explicitly excite the complex (beyond 550nm)? In their current experiment they excite into both the broad complex band and the "narrow" S1 band. If the broad band indeed represents a CT band, then partially exciting it would explain the immediate appearance of the CT state (and the lack of stimulated emission). Having a tunable light source for excitation, it might have been enlightening.

We thank the reviewer for this idea. We have now performed this experiment using the same experimental setup and concentration of samples as used for TRVis in manuscript, with

excitation at 550 nm (below and in Figure S3) and 530 nm (data not shown, but similar behaviour as per 550 nm excitation).

There is no observable change in absorbance upon 550 nm excitation over the experimental timescale of 0.3 – 195 ps, although there is some noise observed around the excitation wavelength. A transient averaged over a 40 nm window around the FMN absorbance maxima also shows no obvious features.

These data can be compared to those measured upon 475 nm excitation (also below), which was measured using the same excitation energies (0.5 µJ). The absorbance at 550 nm is 5-6 times lower than at 475 nm, but the deconvolution of the absorbance spectra (above and new panel if Figure S1) suggests that the "CT absorbance" absorbs roughly as much at 475 as at 550 nm due to its broad nature. Thus, the flavin absorbance is only roughly twice the intensity of the "CT" feature at 475 nm. So, if excitation at 550 nm was going to show any changes in the visible spectral region, then it should have been observable. We have added a note to the manuscript on p.3 that "*Excitation of this [CT] feature at 550 nm leads to no observable TRVis features (Figure S3).*"

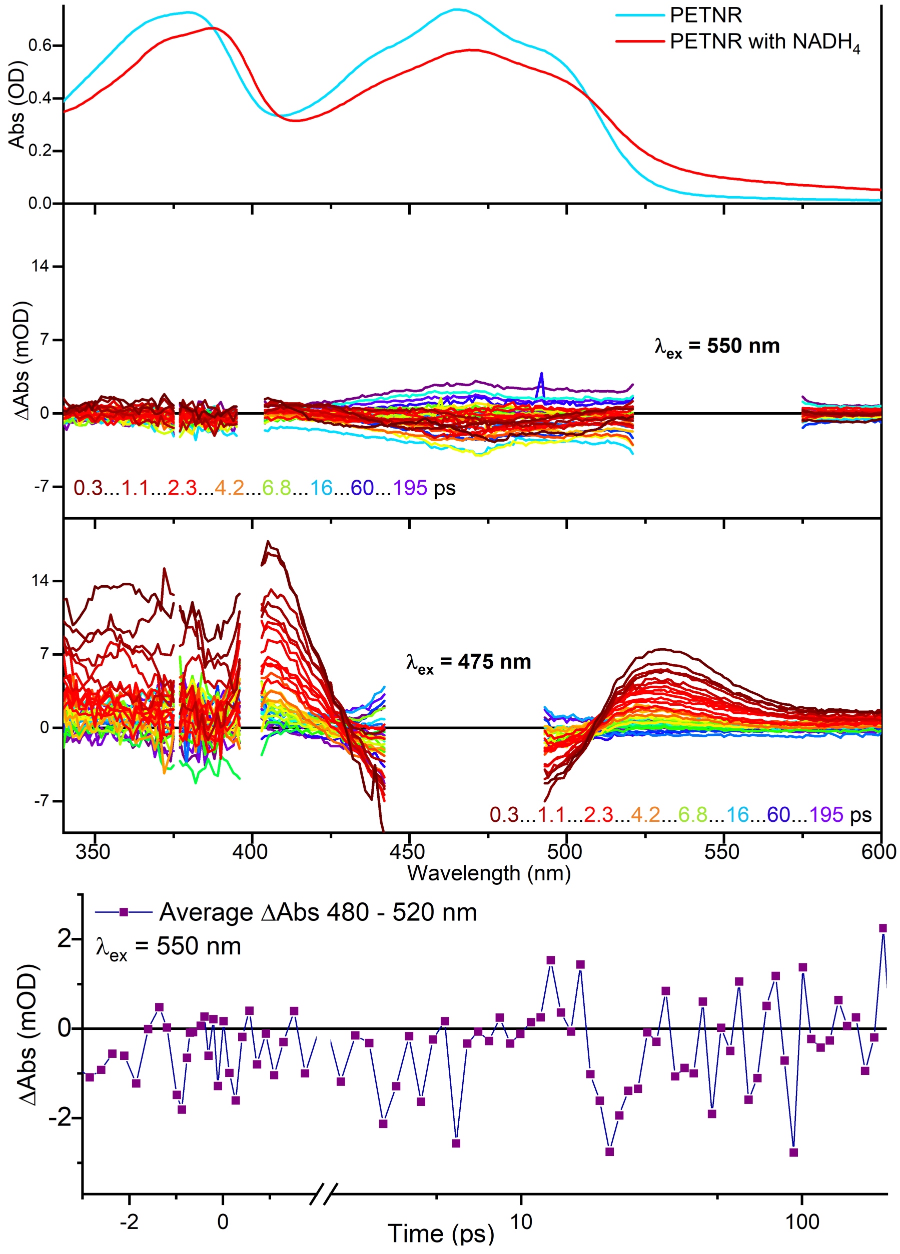

- Have the authors tried to estimate the involved free energy changes using the Weller equation and doing so rationalize the correspondingly fast rates for ET and BET?

Unfortunately, neither the 1-electron reduction potential for PETNR/PETNR semiquinone (as reduction occurs as a concerted 2-electron process) nor the reduction potential for NAD(P)H_4/ NAD(P)$H_4^{+\cdot}$ are known. We are not confident in estimating these values as this will add sufficient error to the predicted rate constant calculated using the Rehm-Weller equation. The electron transfer distance of ~ 3-4 Å (isoalloxazine-nicotinamide ring-ring distance), is very short, so ultrafast eT kinetics are not unexpected.

- This observation "Below 400 nm there is a large positive absorption feature, which does not change in shape until the end of the measurement time frame." is not correct, as would be easily visible from normalized EADS (EADS3 is different in the range < 400nm)

This was poorly described and have rephrased this section on p.3 in response to your comment, below regarding EADS1 assignment.

- It is a pity the authors have not performed emission (ss and tr) experiments, to better understand the nature of the different EADS (in the absence of NADH).

We haven't performed emission experiments here because these are challenging due to the very low intensity, fast lifetime, emission of cofactor-bound PETNR. We have published some TR-emission of PETNR in ref 11. (https://www.sciencedirect.com/science/article/pii/S0006349513011466), and have found that it doesn't provide any additional useful information. The fitted lifetimes are comparable to those observed from TRVis measurements in the current study.

- The assignment of the first lifetime to ET is erroneous in my opinion, as the EADS already completely resembles that of the ionic state (complete lack of stimulated emission). Thus ET has to be faster than the lifetime of EADS1! Also, how is the ET assigned from the "different" structural conformation (EADS2 without NADH)? Does this subpopulation also undergo ET?

For the TRVis data it may be the case that EADS1 corresponds to an eT product, as it has sharp positive features <400 nm previously assigned to the anionic semiquinone, and it is well known that the flavin excited state can be quenched by eT from surrounding residues in protein active sites. However, we don't see significant solvent KIEs on the fitted lifetimes, so these don't appear to report on the formation of the neutral (N5-protonated) semiquinone. We have revised and reordered the text on p.3 to say that in the absence of NAD(P)H_4:
"*The first two evolution-associated difference spectra (EADS) are very similar in shape, and may report on two subpopulations of the protein with different structural conformations and lifetimes.*"
We have now taken care to not assign these EADS to a specific species/redox state of the system with or without NAD(P)H_4, and have changed reference to "EADS 2" later on p.3 to "*These TRvis data*". One possibility is that each subpopulation undergoes eT, but with different kinetics.

The TRIR EADS1 are quite different ± NAD(P)H_4. In Figure S10, we see that only in EADS2 is there a clear negative feature in the approximate location of the amide C=O stretch in NAD(P)H_4 in both ^{15}N PETNR:L FMN and H PETNR:H FMN samples. If this arises from the NAD(P)H_4 cation radical, then it suggests that that EADS1 → EADS2 involves eT from NAD(P)H_4 to FMN.
In the absence of NADP(H)$_4$, the FMN is reductively quenched, likely by eT from active site aromatic residues (Ref 11). This is why the excited state lifetime is much shorter than for free FMN (which is ca. 4ns). This appears to be outcompeted by eT from NAD(P)H_4 when present,

but there is likely to still be an element of eT from aromatic residues observed in EADS1 with $NAD(P)H_4$ is present. On p.5 we now say:
"*This eT occurs in ~1ps and appears to, at least in part, kinetically out-compete reductive quenching from aromatic residues in the active site.*"

- Unfortunately the disentangling of the kinetics (both VIS and IR) is quite hand-wavy and thus remains pretty tentative. E.g. EADS1 in the presence of NADH seems to contain a mixture of features of the oxidized FMN (educt) and the semiquinone (product). If the trivial global fitting approach does not yield meaningful results, the authors should consider alternative means of analyzing their broadband spectra (see e.g. 10.1080/0144235X.2020.1757942).

We thank the reviewer for bringing this useful review of TA/fast fluorescence measurements and data analysis to our attention. In preparing these data for publication we did apply various methods of analysing the data that are described in this paper. We have tried single wavelength and global kinetic analyses, band-shape analysis, SVD etc, some of which we have published before, e.g. in Ref. 11. However, the data collected on these complex biological systems contain many overlapping positive and negative spectral features that are not clearly deconvoluted and the fitted rate/time constants tend to be similar regardless of analysis method. We have settled on a simple global analysis which allows us to identify the most significant features, and then compare with those previously assigned in other works. None of the measurement/analysis techniques used here definitively assign the processes happening, but they all *suggest* that we're seeing eT and back-eT from the $NADP(H)_4$.

Non-scientific changes:

1.) In both the main manuscript file and the Supporting Information, set the title in title case, with the first letter of each principal word capitalized.

Done

2.) Using acronyms in title is discouraged. Please spell out all acronyms in the title of the manuscript and Supporting Information.

We have replaced FMN with flavin and changed $NAD(P)H_4$ to 1,4,5,6-tetrahydro NAD(P)H. If you prefer, this can be expanded to 1,4,5,6-tetrahydro nicotinamide adenine dinucleotide (phosphate)

3.) Shorten the abstract to 150 words or fewer.

Done. It is now 150 words.

4.) Please resize the TOC graphic per journal guidelines (2 in x 2 in) and move to the correct position (on the same page as the abstract).

The ToC graphic has been re-sized and pasted below the abstract.

5.) Remove the section heading(s) throughout the body of the manuscript (you can leave Methods and Abstract headings).

Done

6.) Title must match in three places: (1) manuscript file, (2) supporting information, and (3) ACS Paragon Plus.

Done.

*7.) In both the main file and the supporting information, fix the style of all references to use JPCL formatting (check all references carefully). ***JPC Letters reference formatting requires that journal references should contain: () around numbers, author names, article title (titles entirely in title case or entirely in lower case), abbreviated journal title (italicized), year (bolded), volume (italicized), and pages (first-last). Book references should contain author names, book title (in the same pattern), publisher, city, and year. Websites must include date of access*

Done.

jz-2023-001767.R2

Name: Peer Review Information for "Photoinduced Electron Transfer from a 1,4,5,6-Tetrahydro Nicotinamide Adenine Dinucleotide (Phosphate) Analog to Oxidized Flavin in an Ene-Reductase Flavoenzyme"

Second Round of Reviewer Comments

Reviewer: 1

Comments to the Author

All my concerns have been addressed. I recommend immediate acceptance.

Reviewer: 2

Comments to the Author

The authors have responded to all my questions. I still think, that a more elaborate analysis of the combined TRVis/TRIR data might be beneficial, but I can understand that the authors prefer a more "simple global analysis" approach, which allows only for a rough picture - but at least a picture of this "complex biological system".

One small additional comment on some figure labels in the SI:

Absorbance is a dimension/unitless quantity, thus "Abs (mOD)" or "Abs (OD)" is incorrect.

Author's Response to Peer Review Comments:

Dear Prof Editor,

We are delighted that you are ready to accept your submission after we have made the requested non-scientific changes.

As requested, we have replaced 1,4,5,6-tetrahydro NAD(P)H with 1,4,5,6-tetrahydro nicotinamide adenine dinucleotide (phosphate) in the title and have uploaded revised manuscripted and SI files containing the new title. We have not made any additional changes.

Reviewer 2 commented:

“Absorbance is a dimension/unitless quantity, thus "Abs (mOD)" or "Abs (OD)" is incorrect.”

We have not changed this as the use of mOD units is common in the transient absorption community as it denotes that the measured absorbance has been divided by 1000. As an example, this notation is used

in Figures 4, 18, 25, 32, 55 in the review paper this reviewer cited in their original (10.1080/0144235X.2020.1757942).

Regards,

Sam
